# How Temporal Predictability of Threat and Action Preparation Affect Defensive Freezing Responses

**DOI:** 10.1111/psyp.70278

**Published:** 2026-03-20

**Authors:** Alina Koppold, Mana R. Ehlers, Alexandros Kastrinogiannis, Felix H. Klaassen, Karin Roelofs, Tina B. Lonsdorf

**Affiliations:** ^1^ University of Bielefeld Biological Psychology and Cognitive Neuroscience Bielefeld Germany; ^2^ University Medical Center Hamburg‐Eppendorf Department of Systems Neuroscience Hamburg Germany; ^3^ Department of Neurology Max Planck Institute for Human Cognitive and Brain Sciences Leipzig Germany; ^4^ Radboud University Donders Center for Cognitive Neuroimaging Nijmegen the Netherlands

**Keywords:** action preparation, freezing‐like behavior, psychophysiology, threat imminence, unpredictability

## Abstract

Defensive responses to threat are critical for survival and often involve freezing‐like behavior, which has been linked to action preparation. However, it remains unclear how this preparatory mechanism is influenced by unpredictable aversive stimuli that are difficult to discriminate or anticipate. In this study, we introduce a modified paradigm to examine how freezing‐like behavior is affected by action preparation under temporal threat predictability, unpredictability, and safety. Using a multimodal approach in a large sample (*n* = 235, 148 female), we assessed postural sway and heart rate as markers of freezing‐like behavior, alongside skin conductance levels, electromyographic startle responses, behavioral performance, and subjective ratings. Behaviorally, participants responded faster but less accurately under threat compared to safety, replicating previous findings. Physiologically, threat conditions were associated with both parasympathetically mediated postural freezing and sympathetically driven increases in skin conductance. Importantly, the temporal (un)predictability of threat did not modulate freezing‐like behavior or skin conductance responses. Startle responses were generally inhibited during all conditions (threat and safety) relative to the inter‐trial interval. These findings indicate that action preparation under threat involves concurrent activation of both parasympathetic (postural freezing) and sympathetic systems (skin conductance), consistent with animal models of freezing. The absence of modulation by temporal threat unpredictability may be indicative of a generalized defensive mode that prioritizes readiness over specificity.

## Impact Statement

This study introduces a modified paradigm to investigate action preparation under varying threat predictability conditions using a large human sample and multimodal psychophysiological measures. Contrary to prominent theories emphasizing differential responses to predictable versus unpredictable threats, we found no modulation of freezing‐like behavior or sympathetic system activation by threat predictability. Our findings suggest that in humans, action preparation may function as a generalized defensive mechanism, co‐activating parasympathetic and sympathetic systems irrespective of temporal threat predictability.

## 1 Introduction

Exposure to threat elicits coordinated behavioral, autonomic, and endocrine responses that prepare the organism for rapid defensive actions (Signoret‐Genest et al. 2023). It has been proposed that threat proximity and predictability guide anticipatory action preparation and thereby shape the selection of species‐specific defensive responses, as described by the Threat Imminence Continuum Model (Fanselow and Lester 1988; Mobbs et al. 2020).

Across species, a core preparatory defensive pattern is freezing‐like behavior, classically defined in animals as the absence of overt movement—except for respiration—accompanied by concomitant bradycardia (cardiac freezing, Hagenaars et al. 2014; Roelofs et al. 2010; Roelofs and Dayan 2022; Signoret‐Genest et al. 2023). In humans, freezing (‐like behavior) is presumed to optimize threat detection, risk assessment, and action selection, and can be quantified via stabilometric force platforms (postural sway) and electrocardiography (Azevedo et al. 2005; Roelofs et al. 2010). Freezing‐like behavior differs from tonic immobility, which marks a cessation of active defense. It also differs from orienting‐related immobility, typically observed in response to novel or salient stimuli or slowly approaching predators, which tends to habituate more rapidly with stimulus familiarity than attentive freezing (Roelofs 2017). The current literature on human freezing responses and defensive action preparation reveals mixed findings on both postural and cardiac freezing to threat. While several studies report cardiac freezing in response to anticipated threat (De Voogd et al. 2022; Gladwin et al. 2016; Hashemi et al. 2021; Lojowska et al. 2015), others have observed cardiac freezing over time, specifically during the anticipatory period before cue onset (irrespective of threat or safety cue, Hashemi et al. 2019), or cardiac acceleration during threat anticipation (Wendt et al. 2017). Similar variability is observed for postural freezing, with reports of threat‐related freezing (Azevedo et al. 2005; Hagenaars et al. 2014; Hashemi et al. 2021; Klaassen et al. 2021; Niermann et al. 2018; Roelofs et al. 2010), or no differences between threat and safety (Gladwin et al. 2016; Hashemi et al. 2019). Hence, despite growing interest in human freezing‐like behavior, threat‐induced freezing results across studies remain heterogeneous, possibly due to variability in task designs, research question, and methodological approaches and require further investigation. Other physiological indices, alongside freezing‐related measures, contribute complementary information about defensive preparation. Skin conductance (SC) primarily reflects sympathetic nervous system activation (Boucsein 2012), whereas the fear‐potentiated startle (FPS) reflex is a cross‐species defensive response measured via electromyographic (EMG) activity of the orbicularis oculi muscle elicited by sudden stimuli (Blumenthal et al. 2005). FPS amplitude reflects stimulus valence and defensive engagement (Kuhn et al. 2020; Lang et al. 1990), while latency has been associated with stimulus intensity (Sjouwerman and Lonsdorf 2019) and neural processing speed of threatening input (Koch 1999). Although defensive behavior has been extensively studied and a range of physiological readouts have been established in humans, only one study (van Ast et al. 2022) to date has concurrently examined postural and cardiac freezing alongside skin conductance and electromyography in a fear‐conditioning paradigm.

In addition, many laboratory paradigms investigating defensive responding investigate threats that are predictable. In contrast, real‐world threats are often unpredictable which makes (defensive) action preparation impossible for the organism. The unpredictability of threat onset may shift the balance between rapid but potentially less accurate and slower yet more accurate defensive response actions (Grupe and Nitschke 2013; Roelofs and Dayan 2022). This trade‐off can be further modulated by factors such as threat timing or probability, which influence how efficiently threat cues are detected and acted upon (Blanchard et al. 2010; Mobbs et al. 2007). In laboratory settings, the extent to which threat timing and probability can be learned within an experimental session is highly task‐dependent. Fixed‐contingency paradigms render threat predictability learnable, whereas paradigms with pseudo‐randomized timing or partial reinforcement (e.g., NPU tasks, Grillon et al. 2004; Schmitz et al. 2011 or the Maryland Threat Countdown paradigm, Cornwell et al. 2025) intentionally minimize threat predictability. NPU paradigms are widely used to examine temporal (un)predictability, yet they typically lack an explicit action‐preparation component, which is critical for linking defensive anticipation to motor readiness. Here, we focus specifically on temporal (un)predictability as a key factor modulating action preparation and defensive responses. To date, studies on temporal threat unpredictability (typically in passive viewing tasks without an explicit action‐preparation component) revealed stronger cardiac freezing to temporally unpredictable than predictable threats (Bowers 1971; Corcoran et al. 2021), while skin conductance and fear‐potentiated startle findings remain rather inconsistent. Specifically, some studies report increased SCL under temporal threat unpredictability (Hur et al. 2020; Löw et al. 2015), whereas others find no differences (Bennett et al. 2018; Grillon et al. 2004; Morriss et al. 2022). FPS findings are similarly mixed, reporting potentiation (Bradford et al. 2014; Carsten et al. 2022; Grillon et al. 2004, 2017; Lago et al. 2018; Moberg et al. 2017), inhibition (Löw et al. 2015; Seo et al. 2023; Wendt et al. 2017), or no differences between conditions characterized by temporal threat un‐ vs. predictability (Bennett et al. 2018; Nelson and Shankman 2011; Qiao et al. 2022). Moreover, the interaction between temporal threat unpredictability, freezing‐like behavior, and defensive action preparation has rarely been examined in humans, particularly not using postural sway measures. Examining this holds promise to enhance our understanding how anticipatory defensive states shape subsequent actions, with postural sway providing a sensitive measure of action preparation.

In summary, the understanding of freezing‐like behavior in humans is limited to date for a number of reasons. This is in part because studies employ different operational definitions of freezing‐like behavior and different experimental paradigms to address different research questions. Next, many laboratory studies rely on passive threat‐anticipation tasks that lack an action component, limiting ecological validity for understanding behavior when rapid decisions under threat are required. Moreover, temporal threat (un)predictability, a variable central to ecologically adaptive defensive control, and action preparation have rarely been examined simultaneously in relation to preparatory freezing in humans, particularly in designs integrating multiple physiological systems.

### 1.1 Present Study and How It Addresses These Gaps

**FIGURE 1:**
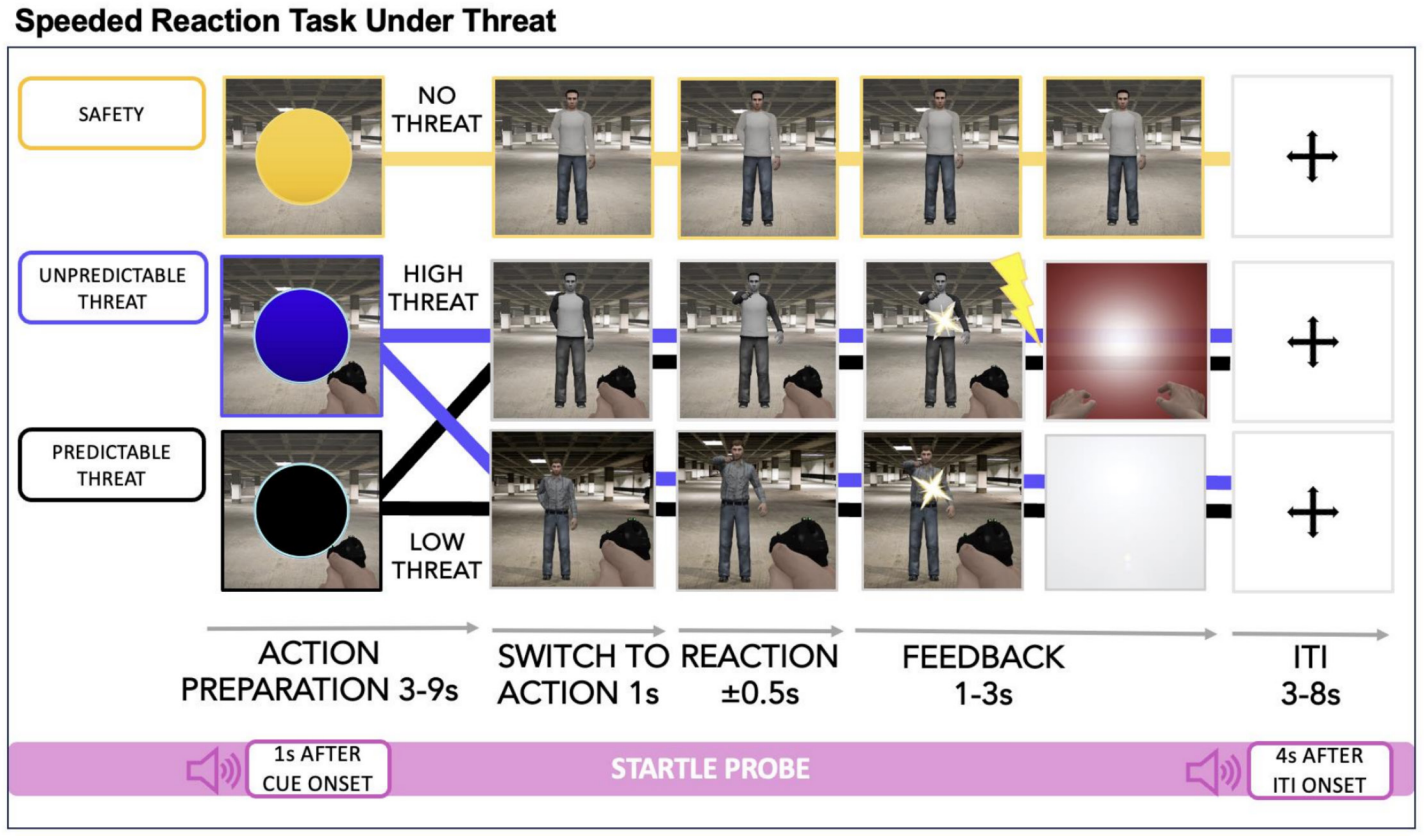
Overview of the GO‐NO/GO Under Threat Task. Each trial begins with an Action Preparation Phase, during which a blinking dot (cue) is displayed for 3–9 s on a static background. Cue color conveys threat level and temporal predictability: Yellow = safe/predictable; black = threat/predictable; blue = threat/unpredictable. Blink rate increases over time in temporal predictable conditions (yellow, black), but remains constant in the unpredictable condition (blue). After the cue, the Action Phase begins: An avatar appears (1 s) and may draw a gun (go trial) or a phone (no‐go trial). Participants must press a button within a limited response window if the avatar draws a gun (defensive action). Failure to respond in time, or incorrect responses, may result in electrotactile stimulation and aversive visual feedback (in high threat condition only). In the final part of each trial, when subjects receive feedback in the threat conditions, either aversive or non‐aversive, examples are shown here: The reddish gradient box in the unpredictable threat condition represents visual aversive feedback (e.g., being “shot” with blood or the arms of a falling/dying subject), whereas the gray gradient box at the end of predictable threat trials illustrates the absence of visual feedback. The mapping of avatars to threat levels (high, low, safe) was counterbalanced across participants for the threat conditions; the safe avatar was constant. Trials end with a jittered inter‐trial interval (3–8 s), during which startle probes were presented in 33% of trials. Startle probes were also presented during the Action Preparation Phase in 33% of trials across all conditions.

The present work directly addresses these gaps by investigating defensive action preparation under temporally predictable vs. unpredictable threat in a paradigm explicitly designed for action preparation. This allows freezing‐like behavior to be studied in an ecologically relevant, action‐oriented context rather than under passive observation. We employ a modified version of the ‘GO‐NO/GO Under Threat Task’ (GUNT) (Gladwin et al. 2016; Hashemi et al. 2019; Tyborowska et al. 2023), in which participants prepare for and execute a rapid motor response under varying threat conditions (see Figure 1 below). Here, temporal unpredictability, operationalized as variable timing of an aversive shock onset during the action‐preparation phase, is used to model the temporal uncertainty inherent in many real‐world threats and to investigate its influence on defensive mobilization (Grupe and Nitschke 2013; Roelofs and Dayan 2022). A multimodal psychophysiological approach is used to comprehensively profile defensive preparation. Specifically, we simultaneously measure postural sway (postural freezing), heart rate (cardiac freezing), skin conductance (arousal), and fear‐potentiated startle amplitude and latency (stimulus intensity Sjouwerman and Lonsdorf 2019; and neural processing speed Koch 1999). This integration of cross‐system indices enables a richer understanding of how autonomic, somatic, and reflexive systems coordinate under threat.

### 1.2 Main Effect of Task

We hypothesize significantly faster and more accurate behavioral reactions under threat as compared to safety during *Action*, as expected from previous work (Gladwin et al. 2016; Hashemi et al. 2019).

### 1.3 Primary Hypotheses

During the *Action‐Preparation* phase, we hypothesize increasing postural and cardiac freezing and increased skin conductance levels (SCL) as threat onset proximity increases, reflecting a main effect of time (with time defined as the decreasing interval until potential threat or safety onset). In addition, we hypothesize stronger postural and cardiac freezing and increased SCL activity under threat (both predictable and unpredictable) compared to safety (defined as main effect of cue), and we further expect a cue (predictable threat, unpredictable threat, safety) × time interaction (effect of action preparation). Finally, based on Wendt et al. (2017) & Löw et al. (2015), we expected startle inhibition, that is, reduced startle amplitudes during threat (both predictable and unpredictable) compared with safety, reflecting a main effect of cue. Because the startle response occurs once during anticipation, it was analyzed only for the main effect of cue, not for time.

### 1.4 Exploratory Hypotheses

Additionally, we will explore differences in the timing and duration of cardiac and postural freezing responses, skin conductance levels, and startle responding between temporal predictable and unpredictable threat conditions during the *action preparation* phase. Based on prior findings for cardiac responding (cue × time interaction, Jennings et al. 1970), we expect decreased cardiac and postural freezing with increasing proximity under temporal threat unpredictability as compared to temporal threat predictability. We will explore SCL and startle amplitudes as secondary outcomes without any priori hypotheses. Furthermore, we will explore the role of startle latency during action preparation, which has been recently highlighted as a potential readout for human anxiety research (Pöhlchen et al. 2023). More precisely, shorter startle latencies under threat irrespective of predictability or unpredictability are expected (main effect of cue, Pöhlchen et al. 2023; Sjouwerman and Lonsdorf 2019).

## 2 Method

### 2.1 Participants

The final recruited sample comprised 235 subclinical adults (for details see Results Table 1). Exclusion criteria included: age below 18 or above 50 years, current medication (except oral contraceptives), pregnancy, participation in other drug‐related studies, internal medical conditions (e.g., cardiac arrhythmias), chronic pain, neurological (e.g., Parkinson's disease) or metabolic disorders (e.g., hyperthyroidism), liver or kidney disease, acute infections, and left‐ or mixed‐handedness (due to additional fMRI assessments reported elsewhere). All participants had normal or corrected‐to‐normal vision and provided written informed consent. The study protocol was approved by the Ethics Committee of the General Medical Council Hamburg (PV5808) and conducted in accordance with the Declaration of Helsinki. This study was part of the larger Fear Profiles project (DFG LO 1980/4–1), in which participants also completed a virtual reality‐based approach‐avoidance task (@Kastrinogiannis et al. 2025), fMRI fear conditioning paradigm (results to be reported separately), and questionnaire battery. The current paradigm (GO‐NO/GO Under Threat Task) and the VR task were administered on separate days (Day 1 or Day 2), with order counterbalanced across participants. Data collection took place between 2022 and 2023, during the COVID‐19 pandemic. Participants were required to provide a negative rapid antigen test until March 1, 2023 (subjects 1–177). All participants were financially compensated for their time (80€).

**TABLE 1:** Sample descriptives.

	***N***	***N* = 235** ^a^
**Age**	234	26.66 (5.49)
**Gender**	234	
Diverse		1/234 (0.4%)
Female		148/234 (63%)
Male		85/234 (36%)
**Gaming experience**	230	1.83 (4.98)
**Years of Education**	221	12.39 (1.10)
**Employment**	230	75/230 (33%)
**If Employment no**.	169	
Currently unemployed		11/169 (6.5%)
Doing military or civilian service/voluntary social year		2/169 (1.2%)
Househusband/ housewife		3/169 (1.8%)
In vocational training		4/169 (2.4%)
Pupil		2/169 (1.2%)
Student		144/169 (85%)
Without a job		3/169 (1.8%)
**Trait Anxiety Sumscore (STAI‐T)**	228	39.55 (10.01)
**Depression Sumscore (BDI)**	228	8.79 (8.03)

^a^ Mean (SD); *n*/*N* (%).

### 2.2 Procedures—Experimental Design

All tasks were implemented in PsychoPy (Version 2021.2.3) and presented on a 32‐in. monitor (Samsung GU32T5377AU; Full HD resolution: 1920 × 1080 px; refresh rate 60 Hz), positioned 60 cm from the participant and 146.5 cm from the floor to the lower screen edge. Participants stood on a stabilometric force platform for the experimental blocks, allowing for continuous recording of postural sway. Concurrently, ECG, skin conductance, and EMG startle responses were recorded (see respective subsections below). The experimental protocol proceeded as follows:

1. Pre‐arrival information: Prior to the laboratory visit, participants received the study information sheet via e‐mail to familiarize themselves with the procedure
2. Arrival and consent: Upon arrival, participants provided written informed consent and a saliva cortisol sample (not analyzed here).
3. Sensor application and calibration: Ag/AgCl electrodes for EMG, SC, and ECG were applied. The intensity of electrotactile stimulation was individually calibrated while participants were seated
4. Instruction phase: Standardized on‐screen instructions were presented (for wording see Supplementary). Participants were then asked to verbally summarize the key task rules to the experimenter to ensure comprehension; any misunderstandings were corrected immediately, and instructions were reiterated if necessary.
5. Pre‐experimental ratings: Participants completed baseline subjective ratings while seated.
6. Training phase: Participants performed 8 practice trials (4 predictable threat, 2 unpredictable threat, 2 safety; randomized order) while standing. All cue conditions were experienced in the training phase. Participants could ask additional questions during the subsequent short break.
7. Experimental phase: Comprised 5 experimental blocks (~20 trials/block; ~5 min each; total 100 trials). Trial order was randomized within each block. Across the full sample, 80% of trials lasted 9 s. Blocks were separated by sitting breaks to reduce orthostatic strain. Initially, 3 breaks were scheduled (participants 1–64); from participant 65 onwards, this was increased to 5 breaks to mitigate nausea.
8. Post‐experimental ratings: Upon task completion, participants completed post‐task subjective ratings.
9. Compensation: Participants completing this task as part of Day 2 of the Fear Profiles Study received financial compensation.

#### 2.2.1 Task Description—Adapted GO‐NO/GO Under Threat Task (GUNT)

We adapted the ‘GO‐NO/GO Under Threat Task’ (GUNT) to study freezing‐like behavior during defensive action preparation under threat of shock (Gladwin et al. 2016; Hashemi et al. 2019; Tyborowska et al. 2023) as well as under (un)predictability. The paradigm was implemented in PsychoPy (v2021.2.3). Each trial consists of two main phases: an Action Preparation Phase and an Action + Feedback Phase, followed by a jittered inter‐trial interval (ITI, Figure 1). Our adapted version of the task manipulates both threat level (safe, low threat, high threat) and temporal threat (un)predictability (temporal predictable vs. unpredictable timing).

#### 2.2.2 Action Preparation Phase (3–9 s)

Trials begin with the presentation of a blinking dot cue over a static background image of an underground parking garage (Figure 1). The dot serves both as a threat valence and temporal predictability cue:

– Yellow dot (Safe/Predictable): Predicts safety. Blink rate increases over time, signaling the upcoming appearance of a safe avatar. No action is required.
– Black dot (Threat/Predictable): Predicts a potential threat. Blink rate increases over time, indicating the approaching appearance (temporally) of an either low or high threatening avatar (see Avatar Description below). Action preparation is required
– Blue dot (Threat/Unpredictable): Also signals potential threat of the approaching appearance of either a low or high threatening avatar, but blinks at a constant rate, providing no temporal information. Trial durations vary, introducing uncertainty. Action preparation is required.

Importantly, the likelihood of encountering the shock‐wielding avatar is *p* = 0.50 in the predictable and unpredictable timing conditions (black and blue dot). Eighty percent of trials in each condition are long trials (9 s) to allow for physiological freezing responses (e.g., heart rate deceleration) to manifest. The remaining 20% are filler trials: medium (6 s, 10%) and short (3 s, 10%) durations are randomly distributed to maintain trial length unpredictability. Startle probes (11 per condition, 33% of trials) are randomly presented during this phase to assess startle modulation and were administered in trials of all durations (3 s, 6 s, and 9 s).

#### 2.2.3 Action Phase (1 s)

After the cue, the dot disappears and is replaced by an avatar that corresponds to the preceding cue:

– Safe avatar (yellow dot): Wears a white shirt and stands still. No threat or action required.
– Low threat avatar (blue or black dot): If it draws a gun, participants never receive an aversive electrotactile shock or visual feedback (a “blood” effect).
– High threat avatar (blue or black dot): If it draws a gun, participants receive an aversive electrotactile shock along with visual feedback (a “blood” effect).

Each threat‐related avatar may draw either a gun (go trial) or a cell phone (no‐go trial). Participants must respond to gun draws by pressing a button within a dynamically adjusted time window (initially 500 ms), simulating self‐defense. To equate the amount of punishment feedback across participants, an adaptive algorithm (Gladwin et al. 2016; Hashemi et al. 2019) adjusted the reaction‐time window so that punishment occurred in approximately 50% of high‐threat trials. No response is required for cell phone trials. In high threat avatar trials, incorrect responses (e.g., failing to shoot a gun‐drawing avatar or shooting a phone‐drawing avatar) are followed by punishment feedback, including shock and aversive visuals (see Figure 1 for examples). To maintain consistent punishment exposure across participants, the time window was adjusted dynamically (e.g., shortened to 200 ms if shocks were too infrequent, or extended up to 1000 ms if too frequent), as described in prior studies (Gladwin et al. 2016; Hashemi et al. 2019). Notably, no punishment was administered for false alarms to the safe or low‐threat avatars.

#### 2.2.4 Inter‐Trial Interval (ITI, 3–8 s, Mean = 6 s)

Each trial ends with a jittered ITI (black fixation cross on white background). Startle probes are presented in 33% of ITIs to capture baseline startle responses. In the Training phase, the ITI between consecutive habituation trials was fixed at 3 s.

#### 2.2.5 Pre‐ and Post‐Experimental Ratings

Participants provided pre‐experiment ratings (0–100 visual analogue scale) of perceived threat predictability for each of the three cues (dots) and three avatars (one safe avatar with no object, and both low‐threat and high‐threat avatars each presented with three different features (gun, mobile phone, or no object)). Post‐experiment ratings captured subjective emotional experience during the experiment across 22 emotions, aversiveness of shock and visual feedback, and gaming experience (see Figures S4 and S5). All rating questions are provided in the Supplementary Section Pre‐ and Post‐Experimental Rating Questions. To collect these ratings, participants provided their answers on a visual analogue scale ranging from 0 (not at all) to 100 (extreme), by sliding a pointer across the scale with the mouse and clicking to confirm. The stimulus and the rating scale were presented simultaneously to facilitate recall and accurate evaluation. Cursor position was centered at 50 at the start of each scale. Reaction times for all ratings were recorded.

### 2.3 Experimental Material and Stimuli

#### 2.3.1 Electrotactile Stimulus

Participants received three 2 ms electrotactile square‐waves on the back of the right hand (inter stimulus interval, ISI: 50 ms) generated by a Digitimer DS7A constant current stimulator (Welwyn Garden City, Hertfordshire, UK) and delivered through a 1 cm diameter platinum pin surface electrode (Speciality Developments, Bexley, UK) fixed between the metacarpal bones of the index and middle finger. The experimental amplitude was calibrated by using a standardized stepwise semi‐automated procedure aiming at an unpleasant, but still tolerable level rated by the participants between 7 and 8 on a scale ranging from zero to 10. More precisely, prior to the experimental task, electrotactile stimulation to the right hand was individually calibrated in two steps. First, the perception threshold was determined by delivering brief stimuli of gradually increasing intensity (0.10 mA increments) until the participant verbally reported detection. Second, the unpleasantness threshold was established by increasing stimulation intensity in 0.5 mA steps and asking participants to rate each stimulus on a numerical rating scale from 0 (“harmless”) to 10 (“unbearable”). The calibration aimed for a target rating of 7 (“very uncomfortable but tolerable”) (Hashemi et al. 2019, 2021). This individually determined intensity was used for all experimental threat trials. Full calibration instructions as presented to participants are provided in the Supporting Information in both German and English.

#### 2.3.2 Auditory Stimulus

White noise burst (i.e., startle probe) at 95 dB was presented simultaneously on both ears via headphones (Sennheiser HD 206, Wedemark, Germany). Startle probes were administered in six trials within the Training phase to achieve a robust baseline startle reactivity (Blumenthal et al. 2005, i.e., startle habituation). During the experiment, probes were presented 4 s after ITI onset in 33 trials and 2 s after anticipation phase onset in 33 trials.

### 2.4 Data Acquisition and Processing

#### 2.4.1 Electromyographic Activity (EMG)

EMG startle was measured below the right eye by using two Ag/AgCl electromyogram electrodes (6 mm sensor diameter, 13 mm outer diameter) placed over the orbicularis oculi muscle and one placed on the participants' forehead as a reference. EMG data was amplified using a V‐AMP 16 amplifier system (16 channel and 2 AUX channel, Brain Products GmbH, Munich, Germany) and sampled with a gain of 5000 at 1000 Hz using Brain Vision Recorder (version 1.23.0001, Brain Products GmbH, Munich, Germany). Offline, raw EMG data were band‐pass filtered between 28‐Hz high‐pass and a 400‐Hz low‐pass, slope 4, and a 50‐Hz notch filter, rectified, and integrated (averaged over 9 ms) using Brain Analyzer software (Version 2.2.0, Brain Products, brainproducts.com). In segmented epochs (100 ms prior to and 500 ms after stimulus onset), EMG data were scored semi‐manually (i.e., computer‐assisted) as foot to peak (20‐150 ms post startle probe onset) according to published guidelines (Blumenthal et al. 2005). Raw data were t‐transformed within each subject [50 + 10*((amplitude‐mean_subject)/sd_subject)] in line with Blumenthal et al. (2005). Responses that were confounded by a blink occurring up to 50 ms before startle probe administration, recording artifacts, or excessive baseline activity (defined as elevated pre‐stimulus EMG activity up to 50 ms before startle probe administration e.g., spontaneous blinks or muscle tension, determined by visual inspection) were scored as missing values (for both amplitude and latency). When subjects showed more than two thirds missing (i.e., recordings confounded by electrode detachments or responses beyond the sampling window) or zero responses across all conditions, they were excluded as non‐responders (Lonsdorf et al. 2019). Zero responses were defined as no detectable EMG blink response. In total, there were 25 individuals identified as nonresponders. Robustness analyses were conducted by including and excluding non‐responders (see details below), which demonstrated no differences in outcomes. Specifically, all significant results remained significant, and all non‐significant results remained non‐significant (data not shown).

#### 2.4.2 Cardiac Responding (Cardiac Freezing)

Cardiac responding was acquired at a sampling rate of 1000 Hz using a V‐AMP 16 (16 channel and 2 AUX channel, Brain Products GmbH, Munich, Germany) and recorded using Brain Vision Recorder (version 1.23.0001, Brain Products GmbH, Munich, Germany) using two passive electrodes (6 mm sensor diameter, 14 outer diameter), with one positioned on the left fifth intercostal space of the thorax and the other (reference electrode) on the right lumbar region. The data were first bandpass filtered with a 1–30 Hz, slope 4 (Mueller et al. 2019). R‐Waves were then detected from the ECG data semiautomatically with the ‘EKG Markers’ solution in the Brain Vision Analyzer Software and visual inspection afterwards with manual correction if necessary, and removal of nonusable data e.g., premature systoles, excessive movement artifacts, Mueller et al. (2019). Next, exported RR‐intervals were converted to HR (in beats per minute) in R Studio (formula: 60000/RR‐interval). To conduct within‐trial analyses over time, we divided the IBI time series into 1‐min epochs referenced to trial onset (blinking dot, Mueller et al. 2019). A baseline correction was performed which averaged the IBI in a time window of 1 s prior to trial onset (Mueller et al. 2019). IBIs were log‐transformed to normalize the distribution. The normal distribution of raw data was tested before and after data transformation with the Shapiro Wilk test.

#### 2.4.3 Postural Body Sway (Postural Freezing)

Using a custom‐built stabilometric force platform [built at the Donders Institute, Nijmegen; as in Niermann et al. 2015; Niermann et al. 2017] of 50.6 × 50.6 cm, the participants' task‐induced postural sway was assessed. At each corner, four pressure sensors enabled the recording of a time series of resistance changes due to a participant's dynamic body posture. Participants stood in a relatively stable position with feet approximately 70 cm apart, and were instructed to stand as still as possible. The platform was calibrated using a 32 kg weight kettlebell (Hastings) before each test session. Posturographic sway was recorded using Python (version 3.6.6) with a sampling rate of 100 Hz and preprocessed and analyzed with R (Version 4.2.2, R Core Team (2022)). For each subject, the mean position of the center of pressure (COP) in the anterior–posterior (AP) and medial‐lateral (ML) direction was recorded per sample point. First, the COP data were bandpass filtered with a low‐pass of 10 Hz and a high‐pass of 0.01 Hz in line with (Gladwin et al. 2016; Hashemi et al. 2019; Niermann et al. 2017). Second, excessive baseline activity (i.e., outliers) was identified according to the following procedure (Gladwin et al. 2016; Hashemi et al. 2019): each data point X of a trial was compared to the overall mean of the trial and identified as an outlier if X was greater than two standard deviations of the trial mean. Next, if more than 50% of the data points within one trial were identified as outliers, the trial was excluded (as described in Supplementary Klaassen et al. 2021). For each trial the standard deviation of the COP in AP direction (COP_AP) was calculated and used as an indicator of the overall change in body movement, i.e., variability in body sway. In line with Roelofs et al. (2010), these standard deviations were then averaged for each condition. In order to precisely locate freezing patterns along the temporal and spatial continuum of threat imminence, the standard deviation of the COP_AP for each 1 s bin within the threat anticipation phase of each trial was extracted. Note that lower scores on postural sway indicate reduced body mobility, and hence, an increase in postural freeze. The normal distribution of raw data was tested before and after data transformation with the Shapiro Wilk test.

#### 2.4.4 Skin Conductance

SC was measured via self‐adhesive Ag/AgCl electrodes (55 mm) which were placed on the palmar side of the left hand on the distal and proximal hypothenar. Hands were cleaned with warm water and without soap. SC was amplified using a V‐AMP 16 amplifier system (16 channel and 2 AUX channel, Brain Products GmbH, Munich, Germany) and recorded constant at 1000 Hz with a gain of 5 μΩ using Brain Vision Recorder (version 1.23.0001, Brain Products GmbH, Munich, Germany). According to published guidelines (Boucsein 2012), data were downsampled offline to 10 Hz. The minimum threshold of SC level was ≤ 0.01 μS (Boucsein 2012). Both tonic skin conductance level (SCL) and phasic skin conductance responses (SCRs) were analyzed, as they index complementary aspects of sympathetic nervous system activity: SCL reflects slow‐changing tonic arousal across sustained periods, whereas SCRs quantify transient, event‐related responses to specific stimuli (here: startle probes, Boucsein 2012).

##### 2.4.4.1 Level

A baseline correction was performed which averaged the SC level in a time window of 1 s prior to trial onset for skin conductance levels in the action preparation phase (0–9 s, see Figure 1) to account for individual differences in skin conductance levels (Kriklenko et al. 2024). To conduct within‐trial analyses over time, we divided the SCL time series into 1‐min epochs referenced to trial onset. When subjects showed more than two thirds missing of trials (i.e., recordings confounded by electrode detachments or responses beyond the sampling window) or zero responses across all conditions, they were excluded as physiological non‐responders (Lonsdorf et al. 2019). Zero responses were defined by response amplitudes ≤ 0.01 μS. Trials with startle probes presentation during the threat anticipation phase were removed as startle probes might modulate the skin conductance levels (De Haan et al. 2018). Additionally, trials that were terminated earlier by button click were removed in line with (Hashemi et al. 2019).

##### 2.4.4.2 Response

For the description of the SCR analysis method see Supporting Information.

#### 2.4.5 Drop Outs Data Quality

Five participants did not show up on the second day of the Fear Profiles Study and did not undergo the Speeded reaction task under threat. Furthermore, 26 individuals were excluded from analysis due to dizziness (standing on the platform for a long time) or insufficient data quality. For startle responding, only 170 datasets (from 235) could be utilized due to an artifact in the reference electrode. For postural sway, 207 datasets were analyzed; for heart rate responding, 184; for skin conductance level, 210; and for skin conductance responding, 216.

### 2.5 Statistical Analyses

Using R and RStudio (Version 4.2.2, R Core Team (2022), R Core Team (2022)). Analyses were performed with linear mixed effects models using the package *lme4* in R Studio (Version 4.2.2). Subsequently estimated marginal means were calculated with the package *emmeans*. The same package was used for post hoc pairwise comparisons.

1. Main Effect Of Task (*Action Phase*): In line with Hashemi et al. (2019), behavioral data comprising response accuracy (shoot vs. withhold) and reaction times (RT) were analyzed to determine whether the presence of threat modulated task performance. LMEMs were implemented to test for differences between high‐threat and low‐threat trials (independent variable) on both RT and accuracy (dependent variables). Importantly, the low‐threat avatar is not the safe avatar, as no responses were required in this control condition. Moreover, this analysis focuses solely on the action phase (avatar displayed), independent of anticipation phase conditions (predictable and unpredictable threat, safety). To further examine combined effects of threat and action type, models included the interaction between avatar threat level (high vs. low) and action type (shoot vs. withhold) with accuracy as the dependent variable. This resulted in four experimental conditions (low‐threat avatar‐shoot, low‐threat avatar‐withhold, high‐threat avatar‐shoot, and high‐threat avatar‐withhold). All models contained random subject intercepts.
2. Primary and Exploratory Hypotheses (*Action Preparation Phase*): Note that trials with durations shorter than 9 s (20% of all trials) were excluded from the psychophysiological analyses. The main effect of cue condition (temporally predictable threat, unpredictable threat, and safety) was analyzed using LMEMs. For each dependent variable (postural sway, cardiac responding, skin conductance level, and startle responding) cue condition was entered as an independent variable, with random intercepts for subjects [model: lmer(dependent_variable∼cue_condition + (1|Subject))]. To examine interactions between cue condition and time (defined as the second within the action preparation phase), additional models were specified with time as an independent variable and its interaction with cue condition [model: lmer(formula = dependent_variable∼cue_condition * time + (1|Subject))]. All models contained random subject intercepts. Post hoc pairwise comparisons were conducted using Wilcoxon signed‐rank tests, and *p*‐values were adjusted for multiple comparisons using the Tukey method.

The alpha level for statistical significance was set at 0.05 (two‐tailed) for all analyses. For data analyses and visualizations as well as for the creation of the manuscript, we used the following R packages.^1^ Data and code are available on Zenodo (doi: 10.5281/zenodo.15111976).

### 2.6 Questionnaires

The Spielberger Trait Anxiety Scales (STAI, Spielberger, 2012) is a measure intended to assess trait negative affect, or personal characteristics related to anxiety, consisting of 20 items rated on a 4‐point Likert scale. The STAI yielded excellent internal consistency (*α* = 0.90) as assessed by Cronbach's alpha and moderate to excellent test–retest reliability coefficients (Barnes et al. 2002). However, the STAI has been criticized for lacking specificity to measuring anxiety ‐despite its name‐ as indicated by poor convergent validity (Knowles and Olatunji 2020) and a strong overlap with depression inventories (Bados et al. 2010). Crucially, although recommendations for cut‐off scores to indicate clinical anxiety are available in the current literature (e.g., ≥ 52; Wiglusz et al. (2019)), there is still no consensus on currently proposed cut‐off scores (see Womble et al. 2021). STAI data for seven participants were not recorded. The Beck Depression Inventory (Beck 1961) is a tool for assessing symptom severity of depression, consisting of 21 items rated on 4 or 5 point Likert scales. The BDI‐II has demonstrated strong internal consistency (*α* = 0.91) and test–retest reliability among healthy samples of adolescents and adults, as well as adult clinical inpatients (Beck et al. 1988; Storch et al. 2004; Wang and Gorenstein 2013). Additionally, it shows acceptable sensitivity and specificity for identifying individuals diagnosed with clinical depression, confirming its usefulness for diagnostic purposes (García‐Batista et al. 2018; Gomes‐Oliveira et al. 2012; Kojima et al. 2002; Segal et al. 2008). BDI data for four participants were not recorded. Internal consistency (Cronbach's α) for the present sample was *α* = 0.92 for the BDI (21 items) and *α* = 0.92 for the STAI (20 items).

## 3 Results

### 3.1 Sample Descriptives

The total recruited sample (without exclusions) is described in Table 1 below. Of note, the study started in November 2021 during restrictions imposed by the COVID‐19 pandemic (i.e., wearing masks, only vaccinated individuals were tested until first of march 2023 (subject 1–177) due to regulations by the university) and ended in October 2023.

### 3.2 Manipulation Check

**FIGURE 2:**
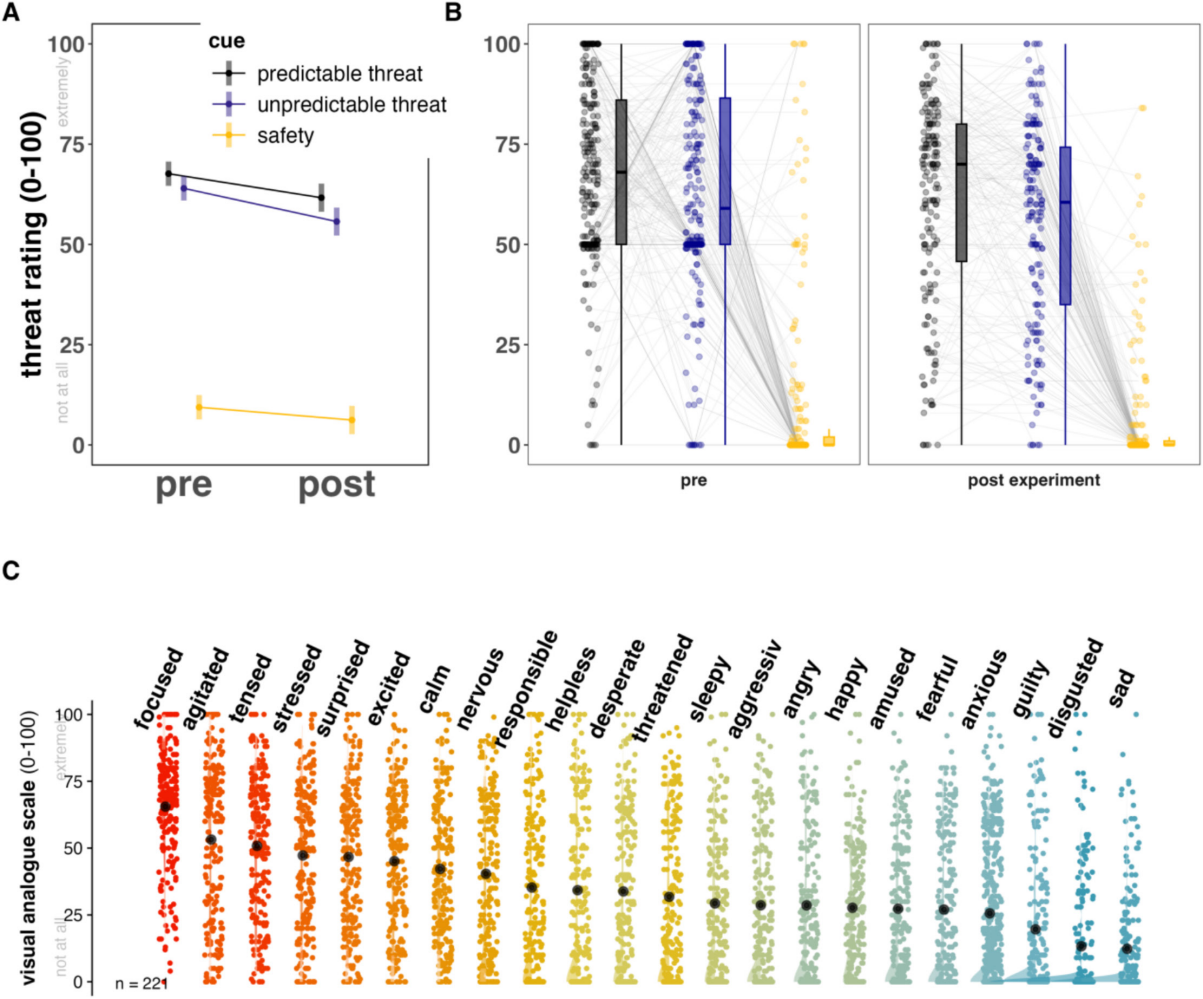
Pre and Post‐experimental threat rating based on a visual analogue likert scale ranging from 0 (not at all threatening) to 100 (extremely threatening) for each condition are depicted with estimated marginal means with 95% confidence intervals (A) and boxplots with line connected dots illustrating threat ratings of each individual for the three cue conditions (B). Post‐experimental ratings of the subjective experience during the task are illustrated using colored density plots accompanied by dots representative for each individual. Emotions appear in descending order based on the sample mean of experienced threat (black dots, C).

First, to verify successful threat manipulation in the action‐preparation phase (see Task Figure 1), we compared pre‐ and post‐experimental threat ratings of cues. As expected, there was a significant main effect of cue, (*F*(2, 999) = 747.64, *p* < 0.001; temporal predictable threat > unpredictable > safety, all pairwise comparisons *p* < 0.001) and a significant main effect of time of assessment (*F*(1, 1123) = 18.81, *p* < 0.001; pre‐task > post‐task), indicating that ratings decreased over the course of the experiment for both threat cues (Figure 2A). No significant cue × time interaction was observed (2, 999) = 1.23, *p* = 0.29. Threat ratings in the temporal predictable and unpredictable conditions were similar in magnitude but showed considerable inter‐individual variability (Figure 2B). Notably, a subset of participants rated the safety cue as moderately to highly threatening, with a few giving ratings at the upper end of the scale, indicating possible generalization of threat to the safe condition. To further verify successful threat manipulation we asked participants to provide threat ratings to the avatars before and after the task. During the action phase high threat avatars were rated as more threatening than low threat or safety avatars (*F*(6,1407) = 45.68, *p* < 0.001; for details see Figure S4). Reaction times of threat ratings of the avatars revealed no significant difference (all *p*'s > 0.25, data not shown). In addition, we post‐experimentally assessed the emotions during the task. As expected, on average, individuals reported high levels of emotions associated with action preparation, e.g., feeling focused (mean = 65.49 ± 21.93) or agitated (mean = 53.20 ± 28.73) but low levels of negative affect, e.g., disgusted (13.29 = 13.08 ± 22.88) or sadness (12.35 = 13.09 ± 19.81, see Figure 2C).

### 3.3 Main Effect of Task

#### 3.3.1 Threat Modulated Behavioral Performance During Action‐Phase

**FIGURE 3:**
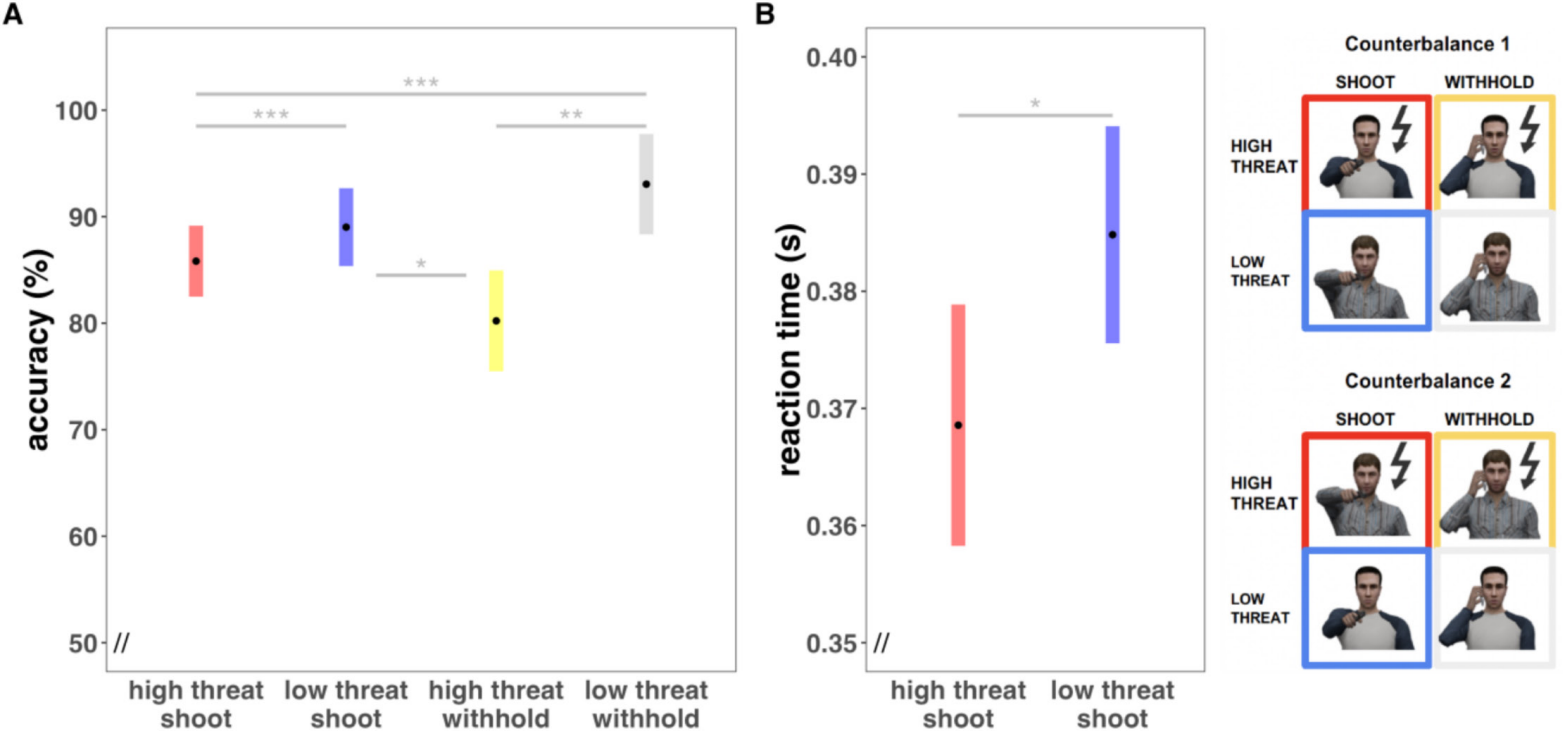
Estimated marginal means with 95% confidence intervals depict the behavioral performance during the action phase of the task indicated by action accuracy (A) and reaction time (B). In line with Hashemi et al. 2019, RT analyses were only conducted for avatars drawing a gun and the means included only correct responses within the response window of 200–500 ms. Binary shooting accuracy (correct vs. incorrect) analyses involved only trials that were not titrated in terms of reaction time window by an algorithm to keep the amount of punishment feedback (threat of shock) comparable across subjects. (*p < 0.05, **p < 0.01, ***p < 0.001).

We aimed to replicate previous reports of improved behavioral performance under threat indicated by faster reaction times at the expense of reaction accuracy (correct vs. incorrect button press) as the main effect of task (Gladwin et al. 2016; Hashemi et al. 2019, 2021) in the “reaction” phase of the task in which participants had to either press a button when confronted with an avatar drawing a gun or withhold when the avatar was drawing a phone (see Task Figure 1). Our results replicated indeed faster reaction times at the expense of accuracy under threat. More precisely, linear mixed effects models revealed a main effect of avatar (*F*(3,284) = 7.79, *p* < 0.001, see Figure 3A) with slightly but significantly higher accuracy (correct button press vs. incorrect) in trials without threat of shock (low threat shoot: mean = 88.44%, SD = 21.19%; low threat withhold: mean = 97.13%, SD = 12.43%) as compared to trials with threat of shock (high threat shoot: mean = 85.32%, SD = 25.01%; high threat withhold: mean = 84.45%, SD = 23.92%, for pairwise comparisons, see Table S1). Similarly, a significant main effect of avatar was observed for reaction time (*F*(1,439) = 6.21, *p* = 0.01, see Figure 3B,C) with significantly faster reactions to avatars associated with threat of shock (mean = 0.37 ms, SD = 0.08 ms) as compared to no threat of shock (mean = 0.38 ms, SD = 0.08 ms). Note that reaction times to low and high threat withhold trials are not included in these analyses as no shooting action is required in these conditions.

### 3.4 Dynamic Modulation of Psychophysiological Responding During Action Preparation *

#### 3.4.1 Postural Sway (Postural Freezing)

**FIGURE 4:**
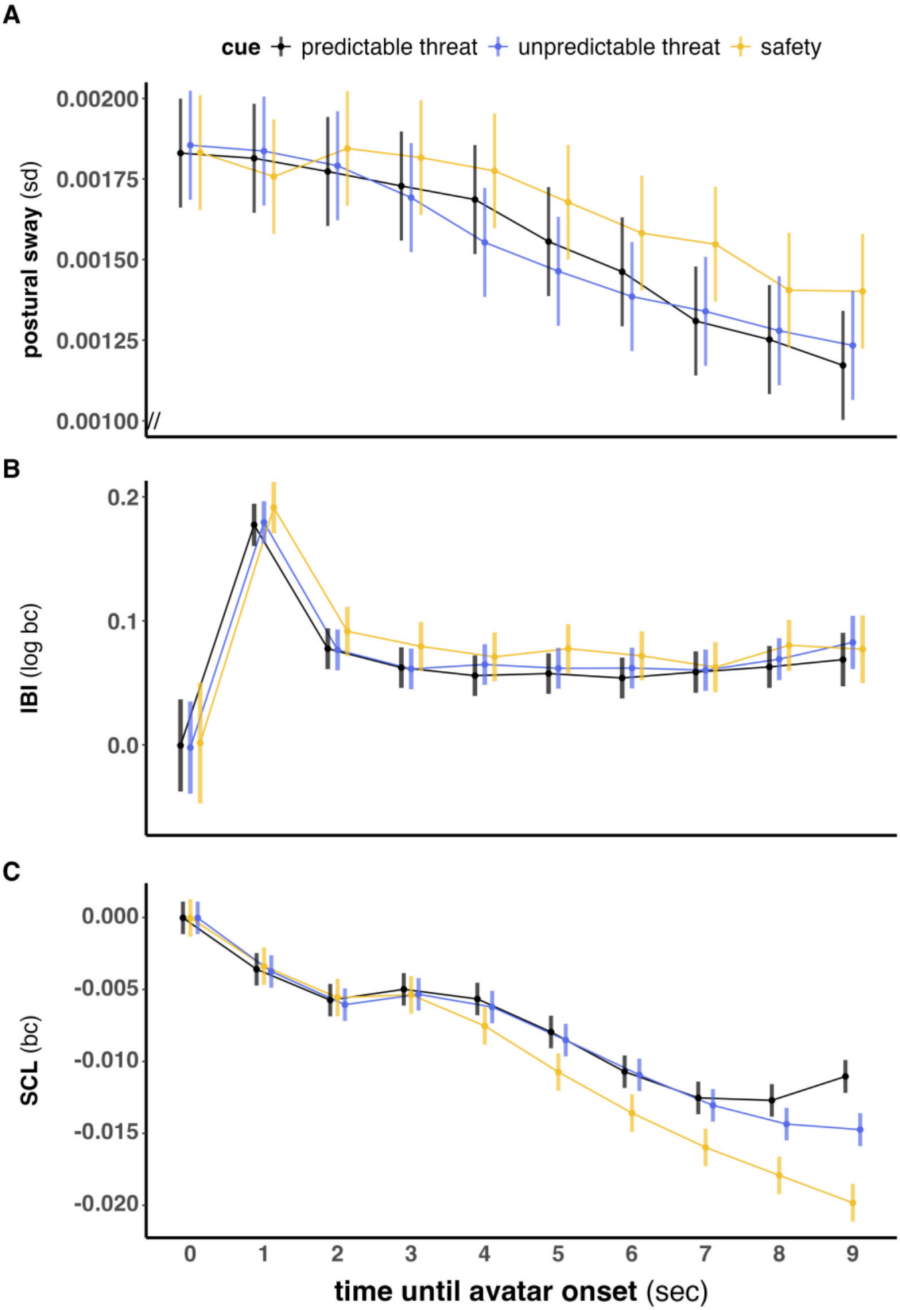
Estimated marginal means with 95% confidence intervals illustrate the physiological responses during the action preparation phase for postural sway (A), cardiac responding (B), and skin conductance levels (C). Note that these analyses are based exclusively on long trials (9 s). bc, baseline‐corrected; sd, standard deviation.

As expected (Gladwin et al. 2016; Hashemi et al. 2019), the linear mixed‐effects models revealed a significant main effect of cue on postural sway (*F*(9,163,533) = 109.36, *p* < 0.001), with reduced sway under threat anticipation (i.e., increased postural freezing). Both temporally predictable and unpredictable threat conditions were associated with stronger postural freezing than safety, but did not differ significantly from each other at the overall level (all *p* ≥ 0.93; Figure S2A). There was also a significant main effect of time (*F*(2,163,534) = 28.71, *p* < 0.001), with sway decreasing over the 9‐s action‐preparation phase. In line with previous work, freezing‐like behavior was operationalized as a reduction in postural sway amplitude and an increase in inter‐beat interval from early to late segments of the action‐preparation phase within a trial, with greater changes expected under threat compared to safety conditions (Roelofs 2017; Roelofs and Dayan 2022). Importantly, the cue × time interaction was significant for postural sway (*F*(18,163,533) = 2.94, *p* < 0.001, Figure 4A), but not for cardiac responding (see below). Postural freezing was stronger in the second half of the preparation phase (seconds 5–9) in both threat conditions relative to safety. Pairwise comparisons showed that the temporal unpredictable‐threat condition exhibited significantly increased postural freezing from second 4 through 9 compared to safety, with the effect descriptively smaller but still significant in the final seconds. Temporal predictable‐threat trials showed significantly greater postural freezing than safety only at seconds 7 and 9. No significant differences emerged between the two threat conditions at any individual time point (all *p* ≥ 0.93; Table S2).

#### 3.4.2 Cardiac Responding (Cardiac Freezing)

There was a significant main effect of cue (*F*(9,181,205) = 83.86, *p* = 0.002), with increased inter‐beat intervals (IBIs; cardiac freezing) under both threat conditions relative to safety (Figure S2B). The main effect of time was also significant (*F*(2,181,227) = 1.72, *p* < 0.001), with IBIs increasing over the course of the preparation phase. However, the cue × time interaction was not significant (*F*(18,181,201) = 0.39, *p* = 0.99; Figure 4B), indicating that the temporal profile of cardiac freezing did not differ across conditions.

#### 3.4.3 Skin Conductance Level (SCL)

A significant main effect of cue was found for average SCL (*F*(9,185,142) = 509.53, *p* < 0.001), with higher levels in both threat conditions compared to safety (temporal predictable threat = unpredictable > safety; Figure S2C). Time also had a significant main effect (*F*(2,185,146) = 87.82, *p* < 0.001), with SCL decreasing across the action‐preparation phase regardless of condition. The cue × time interaction was significant (*F*(18,185,141) = 11.83, *p* < 0.001, see Figure 4C). Post hoc comparisons indicated that SC levels in both temporal predictable‐ and unpredictable‐threat conditions were significantly higher than in the safety condition at several individual mid‐ to late‐phase time bins (≥ + 5 s), up to the end of the action‐preparation phase (see Table S3 for exact time points). Notably, temporal predictable threat exceeded unpredictable threat only in the final second (+9 s), immediately before subsequent action.

#### 3.4.4 Skin Conductance Response (SCR) to Startle Probes

Phasic SCRs to startle probes showed no significant difference between the action‐preparation phase and the inter‐trial interval (*F*(1,12,695) = 1.6, *p* = 0.20), nor between cue conditions (*F*(2,6152) = 0.3, *p* = 0.77; Figure S6). This indicates that startle probes did not modulate SCR amplitudes in this task.

### 3.5 The Association Between Startle Inhibition and Action Preparation

**FIGURE 5:**
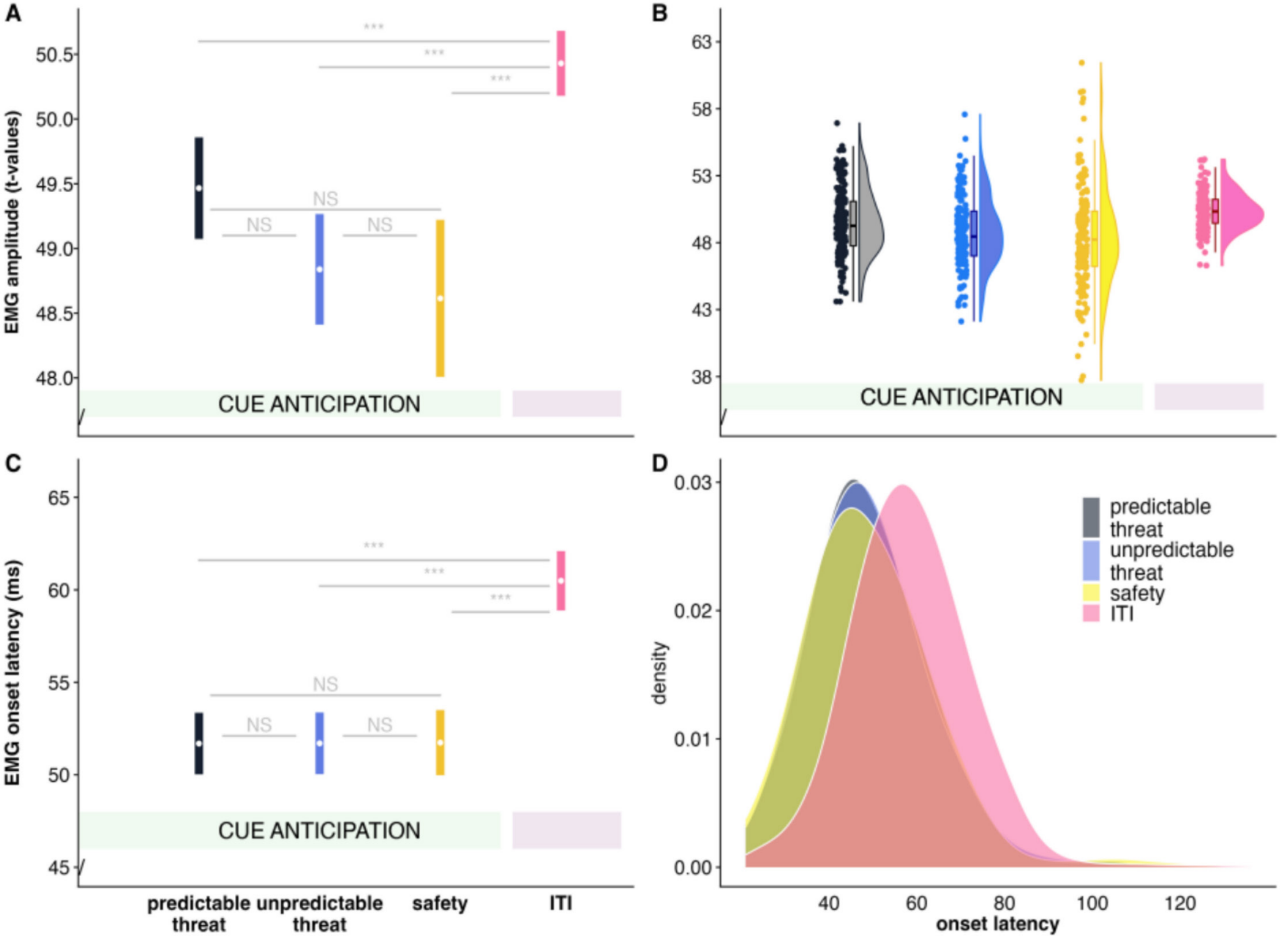
Estimated marginal means (based on t‐transformed amplitudes and raw latencies) with 95% confidence intervals (A) and half violin dot plots with densities and individual data points for each subject for startle amplitudes (B) to probes presented during the action preparation phase (temporal predictable/unpredictable threat, safety) and inter‐trial interval. Note that, during the action preparation phase (temporal predictable/unpredictable threat, safety condition) 10 trials for each condition are available for each subject, whereas in the ITI condition 30 trials for each subject were available. Estimated marginal means (based on raw latencies) and 95% confidence intervals (C) and density plots (D) for EMG startle onset latency to startle probes presented during the action preparation phase (temporal predictable/unpredictable threat, safety) and inter‐trial interval depicting generally earlier onset latencies during action preparation. Note that t‐transformation was performed before outlier removal.

In line with our hypotheses, we observed decreased startle response amplitudes under action preparation in all (threat, safety) cue conditions compared to responses during the ITI (see Figure 5A, main effect of cue; *F*(3,10,205) = 20.36, *p* < 0.001). Yet, contrary to our hypotheses, startle amplitudes were also significantly reduced in the safe cue condition, as compared to those elicited during the ITI (see Figure 5B) and did not differ from those elicited during the cue conditions (i.e., temporal predictable and unpredictable threat, for pairwise comparisons see Figure 5). In addition to decreased startle amplitudes under action preparation, we also observed an earlier startle onset latency (*F*(3,7401) = 391.56, *p* < 0.001, see Figure 5C,D) and startle peak latency (*F*(3,9233) = 4.60, *p* < 0.001) across all cue conditions as compared to the ITI. In sum, these results revealed that the task context was associated with faster and reduced‐amplitude EMG startle responses in both threat trials with action preparation and safe trials without action preparation, with both conditions differing from the inter‐trial interval.

## 4 Discussion

Defensive responding is an essential process ensuring survival across species. Especially first responders (e.g., police officers, firefighters or paramedics), who are required to protect not only themselves but also members of our society are required to train the action preparation mechanism to switch to optimal action under immense stress (e.g., under threat). Importantly, in practice, hazardous situations are often characterized by temporal unpredictability about the proximity of the threat, the timing of a potential attack, and the exact location of the threat, hampering defensive action preparation. Here we investigated defensive action preparation under threat in a large sample of 235 individuals and replicated previous findings of general startle inhibition (Richter et al. 2012; Wendt et al. 2017), increased skin conductance levels (Löw et al. 2015; Wendt et al. 2017), and increased freezing‐like behavior (Hashemi et al. 2019, 2021) during action preparation under threat. Moreover, we investigated whether defensive freezing‐like behavior is modulated by temporal threat (un)predictability. Contrary to our expectations, no differences in psychophysiological responding during action preparation were observed for temporally predictable and unpredictable threat conditions.

### 4.1 Characterizing the Psychophysiological Cascade During Action Preparation in the Face of Threat

Freezing‐like behavior has been conceptualized as a predominantly parasympathetically‐driven “break” on motor and visceral activities which hence contributes to sustained attention and prioritizing bottom‐up or stimulus‐driven activity over top‐down input (Roelofs 2017). To this end, it might serve to facilitate subsequent action (Roelofs and Dayan 2022), which manifests as faster reaction times and higher response accuracy in the face of threat (Wendt et al. 2017). Our results replicated indeed faster reaction times at the expense of accuracy under threat rather than enhanced accuracy (Hashemi et al. 2019). Moreover, our results replicated the expected stronger threat‐induced postural and cardiac freezing (Hashemi et al. 2021) as well as increased skin conductance levels (Löw et al. 2015) during defensive action preparation prior to switching to action. Furthermore, following van Ast et al. (2022) we observed increased threat‐induced postural, but not cardiac freezing and increased skin conductance levels with increasing threat imminence during defensive action preparation as compared to safety (interaction cue × time). This finding aligns with the current conceptualization of “freezing” as a reduction in postural sway over time which is expected to be particularly pronounced under aversive or threatening conditions (Roelofs 2017; Roelofs and Dayan 2022). The absence of threat‐induced cardiac freezing with increasing threat imminence was contrary to our hypotheses, but does in fact align with previous studies which also did not report a time × cue interaction Hashemi et al. (2019), but do report a main effect of cue (Hashemi et al. 2021) and/or time (Hashemi et al. 2019; Klaassen et al. 2021; Merscher et al. 2022; Swiercz et al. 2018; Vila et al. 2007). Here, we also observe both a main effect of time as well as cue. It has been assumed that, for the main effect of time, cardiac freezing becomes stronger over time independent of threat, which is associated with a state of selective, vigilant attention and response preparation (Hashemi et al. 2019). In contrast, there are also reports of threat‐induced heart rate acceleration (cue × time interaction, Wendt et al. 2017) in conditions with increasing threat imminence and an action component as compared to cardiac freezing observed in threat conditions without an action component. The authors suggested threat‐induced cardiac freezing as an “increased orienting response towards external stimuli which only evolves into full cardiac freezing when attention is focused on a predator coupled with motor freezing” (cf. Wendt et al. 2017). In our paradigm, increasing threat imminence was hypothesized to contribute to bradycardia, whereas a rapid increase in the flicker rate of the stimulus (dot) could potentially enhance sympathetic activation, which might counteract or mask this effect. From this point of view, no final conclusions can be made on the role of heart rate responding during defensive action preparation and requires the extension of our paradigm to include threat conditions with as well as without an action component in future studies to replicate the finding of Wendt et al. (2017). Moreover, future work should employ equal average flicker frequencies across conditions and include an additional unpredictable safety condition to directly assess the potential influence of flicker‐rate differences on physiological responses. Furthermore, there are also substantial individual differences in whether an individual shows heart rate acceleration or cardiac freezing in the face of threat (Jaswetz et al. 2025). Hence, future work focusing on (latent) subgroup and/or trajectory analyses would be an interesting next step. As stated earlier, it has been assumed that defensive action preparation is associated with a co‐activation of both parasympathetic and sympathetic branches of the autonomic nervous system (Roelofs 2017). In line with this and previous work, the parasympathetically driven threat‐induced cardiac and postural freezing was accompanied by an increase in sympathetic nervous system reactivity as indexed by attenuated habituation of SCL under threat (Löw et al. 2008, 2015; Wendt et al. 2017). In the literature such heightened sympathetic activity in the face of threat has been linked to enhanced attention as well as facilitation of both perceptual as well as motor processing (Löw et al. 2015; Merscher et al. 2022; Pribram and McGuinness 1975), both of which are processes serving subsequent actions (Roelofs and Dayan 2022). In summary, our results indicate an increased co‐activation of parasympathetic freezing‐like behavior alongside sympathetic nervous system reactivity during action preparation under threat as compared to safety. Prediction and action preparation are central to navigate an ambiguous and uncertain world (Grupe and Nitschke 2013). Crucially however, hazardous situations are often marked by unpredictability regarding the proximity of the threat, the timing of an attack, or the perpetrator's exact location, complicating effective defensive action preparation. Hence, a potential modulation of these response patterns by temporal threat (un)predictability was a key focus of the present work.

### 4.2 Is Unpredictability About the Timing of a Threat Associated With Alterations in the Preparatory Cascade for Defensive Actions?

It has been proposed that the temporal unpredictability of threats disrupts the typical defensive response cascade in a threat‐imminence and (un‐)predictability dependent manner (Fanselow 2022). Contrary to this hypothesis, however, our results revealed no differences in freezing‐like behavior (cardiac and postural) during action preparation under temporal predictable versus unpredictable threats. This finding might suggest that action preparation under threat in humans prioritizes a general state of readiness over the precision or specificity of defensive responses. More precisely, it may suggest that when faced with a threatening situation, the human nervous system may default to a broad, heightened state of arousal, enabling rapid response to potential danger, rather than finely tuning reactions to specific threat features such as the level of temporal unpredictability. Such adaptive mechanisms may reflect evolutionary developments, favoring survival by preparing for immediate action in uncertain and rapidly changing environments, even if it comes at the cost of precise threat discrimination (Mobbs et al. 2015, 2024). Alternatively, it can be speculated that although participants were able to differentiate between these two conditions in terms of threat ratings, the temporal unpredictable condition may have involved temporal predictability unintentionally which may consequently render (anticipatory) responding in both conditions indiscriminable. More precisely, while the temporal threat unpredictability cue did not blink more rapidly as proximity to the action increased, participants may still have used temporal patterns in the sequence to infer temporal predictability (as time passed, the need to react generally increased, even in the unpredictable condition), which could have led to comparable de‐facto temporal predictability across conditions (see Figure 2A). It could be speculated that, hence, differences between the temporal predictable and unpredictable threat trials were more noticeable early in the threat anticipation window. Furthermore, as this study utilized a within‐subject design, participants were exposed to both threat conditions (temporal predictable, unpredictable), which might have facilitated the detection of such inbuilt but unintended temporal predictability.

Interestingly, we observed a significant increase in the activation of the sympathetic nervous system (as indexed by SCL) in the temporal predictable condition as compared to the unpredictable condition in the final second prior to switching to action while both conditions did not differ from each during the 8 s before, a pattern consistent with prior findings (Cornwell et al. 2025; Monat et al. 1972). This may point to increased engagement of attention and motor resources in preparation for imminent actions that are specific for temporally rather precisely predictable action under threat. In startle responding, however, in line with previous findings (Seo et al. 2023), we did not observe significant differences in startle responding (neither in amplitudes or latencies) between conditions of temporal predictable and unpredictable threat. In sum, understanding the mechanisms of action preparation in response to temporal predictable versus unpredictable threats is clinically highly relevant, promising to provide insight into the heightened threat responses seen in anxiety disorders and related psychopathologies, where individuals show exaggerated defensive behaviors and hypervigilance, particularly when facing unpredictable threats. Even though our results did not reveal significant or clear difference in action preparation for temporal threat predictability versus unpredictability indicated by freezing‐like behavior (postural, cardiac) and startle responding, this finding should be replicated and elaborated on by fine‐tuning the experimental design of future work.

### 4.3 Postural Freezing: Measurement Challenges and Impact on Result Variability

While the analysis and operationalization of most measures (HR, startle, SCL) used in this study has been well established for decades (SCL: Boucsein 2012; startle: Blumenthal et al. 2005; HR: Quigley et al. 2024), the measurement of postural sway as an index of freezing‐like behavior in humans is a more recent development (Roelofs 2017; Roelofs and Dayan 2022). Postural freezing is a psychophysiological response characterized by a reduction in body sway, which has been associated with defensive action preparation in the face of threat (Roelofs 2017). Its measurement typically involves the quantification of postural sway dynamics using force platforms or motion capture systems, enabling the detection of reduced movement variability or amplitude as an index of freezing. In particular cardiac and postural freezing have been used in previous work employing similar or closely related paradigms to investigate action preparation under threat. The results from this literature are, however, difficult to integrate due to substantial heterogeneity in operationalization of the key indexes as well as the precise statistical effect taken to indicate freezing‐like behavior as well as unclear empirical evidence. More precisely, in previous work using an identical paradigm, freezing‐like behavior” (i.e., postural and cardiac freezing) was operationalized as (i) general movement reductions over time (i.e., main effect of time) without the need for an additional main effect of threat or an additional threat‐time interaction (Hashemi et al. 2019) or as (ii) a main effect of threat on postural freezing in absence of a significant main effect of time or threat‐time‐threat interaction (Hashemi et al. 2021). Here we conceptualized freezing‐like behavior in a slightly modified paradigm according to recent reviews (Roelofs 2017; Roelofs and Dayan 2022) as (iii) a defensive action preparation response to threats becoming stronger over time (threat × time interaction). Hence, we call for a clear definition, operationalization, and statistical operationalization of freezing‐like behavior to foster and facilitate cumulative knowledge generation in the future (Röseler et al. 2024).

In addition to the question on “how” to measure postural freezing, a related question on “what” freezing‐like behavior (both postural and cardiac) actually measures is equally central. According to current views, freezing‐like behavior in humans is thought to represent a state that serves to facilitate defensive action preparation (Roelofs 2017). However, when reviewing the current literature on freezing‐like behavior (both postural and cardiac), it becomes apparent that an overwhelming majority of studies examining freezing‐like behavior employ tasks without an action (preparation) component, such as those involving emotional pictures or video viewing (Azevedo et al. 2005; Facchinetti et al. 2006; Fragkaki et al. 2016; Fragkaki et al. 2017; Hagenaars et al. 2012; Hagenaars et al. 2014; Hillman et al. 2004; Lelard et al. 2014; Roelofs et al. 2010; van Ast et al. 2022; Stins and Beek 2007; but see Gladwin et al. 2016; Hashemi et al. 2019; Klaassen et al. 2021). Of note, these studies show robust postural and cardiac freezing in conditions with negative pictures as compared to neutral or positive pictures—in the absence of any action preparation. However, it remains possible that action preparation processes are still present but not captured due to the design of these studies. Freezing‐like behavior could, in principle, reflect an implicit form of action preparation even when not overtly required by the task. Future research should consider whether such tasks might still elicit preparatory motor or autonomic processes, even if they are not explicitly measured. Thus, the question arises whether freezing‐like behavior is a proxy for defensive action preparation or primarily a fear‐ and threat‐related response irrespective of action preparation (response to negative pictures). For instance, Löw et al. (2015) observed cardiac freezing responses under the active and passive threat conditions, as well as the active safe condition. However, cardiac freezing in the passive threat condition occurred later than cardiac freezing during both active conditions. Notably, there was no significant difference between the active conditions (safe vs. threat) regarding cardiac responses or timing (Löw et al. 2015). From this, it could be inferred that the action preparation mechanisms indicated by cardiac freezing are rather independent of threat (safe vs. threat cues) but are dependent on threat imminence (threat × time interaction). However, this study (Löw et al. 2015) did not assess postural sway, and hence, the generalizability of findings on cardiac freezing to postural freezing must remain speculative for now. Replicating and extending this study design, including measures of postural sway, could serve as a promising starting point for further exploration of freezing‐like behavior as a proxy for action preparation in humans. Worth mentioning here is that startle inhibition emerges as a particularly promising psychophysiological marker of action preparation. In this study, startle inhibition, characterized by reduced amplitudes and earlier onset latencies, has been observed across conditions alike (safe, temporal predictable threat, unpredictable threat) as compared to responses during the inter‐trial intervals, potentially facilitating subsequent actions. As action preparation was required only in threat trials but not in safe trials, yet startle inhibition was still observed, this suggests that the inhibition effect may not be solely due to action preparation. Instead, it could be influenced by other factors, such as attentional engagement or the general anticipation of events, regardless of whether a motor response is explicitly required. However, the finding of defensive startle inhibition prior to action aligns with previous work (Richter et al. 2012; Wendt et al. 2017) reporting a shift from startle potentiation in conditions without an action component to inhibition in conditions with an action component. Moreover, this pattern is consistent with the observation of defensive startle potentiation in aversive situations without action preparation elements, such as during affective (picture viewing) tasks (Azevedo et al. 2005; Hagenaars et al. 2014; Hillman et al. 2004; Mouras et al. 2015; Noordewier et al. 2020; Roelofs et al. 2010; Stins and Beek 2007) or threat conditioning (Szeska et al. 2021; van Ast et al. 2022), where van Ast et al. (2022) demonstrated a clear link between reduced postural sway and heightened startle responses despite the absence of any action‐preparation requirements. Hence, preparation for action under threat is associated with reduced startle amplitudes but faster onset of startle responses, suggesting it as a potential valuable readout measure for investigating action preparation in humans.

The study's strengths lie in its large sample as well as in combining critical replication effort with novel research questions. In addition, the employed, a multi‐methodological approach incorporating skin conductance level and EMG startle responding adds valuable insights into the action processing cascade under stress beyond traditionally employed measures of postural and cardiac freezing (Hashemi et al. 2019, 2021; Klaassen et al. 2021). Despite these strengths, we acknowledge some limitations. First, the results are based on a predominantly homogeneous sample (e.g., mostly students, approximately two‐thirds female), and were collected under the specific setting requirements of the COVID‐19 pandemic. More precisely, recent evidence suggests that although behavioral performance remains comparable between mask‐wearing and non‐mask‐wearing participants, physiological demands are increased (e.g., elevated heart rate and decreased blood oxygen saturation, Tornero‐Aguilera and Clemente‐Suárez 2021), which might have interfered with our psychophysiological measures. Second, all three Action Preparation conditions spanned the same 3–9 s anticipation window, with 80% of trials (4 out of 5) lasting exactly 9 s. As a result, the majority of trials were identical in duration, which likely reduced the effective degree of temporal uncertainty between conditions (e.g., black vs. blue dot) and may have limited the strength of the temporal uncertainty manipulation. While the present study specifically examined the temporal predictability of threat onset by using a flickering stimulus with varying frequencies and temporal proximity to the threat, future studies may benefit from employing a broader range of trial durations and from exploring other dimensions of threat unpredictability (e.g., perceptual or shock probability) to enhance generalizability (Roelofs and Dayan 2022; Skora et al. 2022). Moreover, the present task differs from many passive, uncontrollable threat paradigms in that punishment outcomes were partly contingent on participants' reaction times, meaning that unpredictability occurred within a controllable operant framework, which may modulate its impact compared to truly uncontrollable threat contexts. In summary, our results demonstrate the conceptually predicted (Roelofs 2017) parasympathetic and sympathetic co‐activation during action preparation under threat, indicated by increased postural freezing and skin conductance levels and highlight startle inhibition as an additional proxy for assessing defensive action preparation. Crucially, our investigations did not reveal differences in defensive action preparation under temporal predictable or unpredictable threat in terms of freezing‐like behavior. This lack of difference in freezing‐like behavior between temporal predictable and unpredictable threats could suggest that defensive action preparation serves as a generalized mechanism, prioritizing readiness over specificity when facing potential threats. However future studies optimized for predictability investigations are needed. Together, our findings refine our understanding of defensive behaviors and emphasize the need for further research to explore how contextual and individual factors might modulate this process.

## Author Contributions

**Alina Koppold:** methodology, conceptualization, investigation, validation, formal analysis, visualization, data curation, writing – original draft, writing – review and editing. **Tina B. Lonsdorf:** conceptualization, methodology, formal analysis, writing – original draft, writing – review and editing, supervision, funding acquisition. **Alexandros Kastrinogiannis:** investigation, data curation, writing – review and editing. **Mana R. Ehlers:** investigation, data curation, writing – review and editing. **Karin Roelofs:** writing – review and editing, methodology. **Felix H. Klaassen:** methodology, writing – review and editing.

## Funding

This work was funded by a grant by the German Research Foundation (Deutsche Forschungsgemeinschaft) to TBL: DFG LO1980/4‐1.

## Conflicts of Interest

The authors declare no conflicts of interest.

## Supporting information


**Data S1:** psyp70278‐sup‐0001‐DataS1.docx.

## Data Availability

The data that support the findings of this study are openly available in Zenodo at https://doi.org/10.5281/zenodo.18713991.
