# Supplementary material for: How Temporal Predictability of Threat and Action Preparation Affect Defensive Freezing Responses
Source: Psychophysiology. 2026 Mar 20;63(3):e70278. doi: 10.1111/psyp.70278 (PMC13004758; doi:10.1111/psyp.70278)
Supplement: Supplementary file 1 — Data S1: psyp70278‐sup‐0001‐DataS1.docx. [file PSYP-63-e70278-s001.docx]

# Appendix

# Supplementary

## Instruction Experiment

**English version**
Dear Participant, You will now find yourself in an underground parking garage setting (image). In this setting, a coloured dot will appear (image). Please pay close attention to these coloured dots. The different colours at the beginning of each trial indicate how close the threat is. They flash/blink at different speeds: blue = unpredictable, black = predictable (the faster the blinking, the closer the threat), yellow = safe / no danger (image). Following this, you may encounter one of two possible attackers in the parking garage (image). The counterpart may be holding either a weapon or a mobile phone: Weapon = threat (image) Mobile phone = no threat (image) If you encounter the person wearing the chequered shirt [shirt colour counterbalanced between participants] and you are either (a) shot by them or (b) shoot them mistakenly when they are holding a mobile phone, you will NOT receive an electrotactile stimulus (image). If you encounter the person wearing the black‑and‑white shirt and you are either (a) shot by them or (b) shoot them mistakenly when they are holding a mobile phone, you will receive an electrotactile stimulus (image). Your task: You can protect yourself from the attacker by pressing a key as quickly as possible to block the shot. If you are too slow, you will be shot and will receive an electrical stimulus to your hand. Note: The man holding a telephone is not a threat, therefore you do not need to protect yourself. If you shoot mistakenly (key press) at a man with a phone, you will receive an electrical stimulus. During the experiment, you will hear tones through the headphones you are wearing. Please now tell the experimenter the task in your own words. You may ask any questions at this point. […] Ready? First, there will be safety questions. You will then begin with a practice block (8 trials), after which you may ask questions again, followed by the experimental blocks. Remain standing calmly on the stabilometric force platform throughout the experiment. Try to keep your left hand as still as possible. […] Thank you for participating in the experiment. You will now answer the final questionnaires.

**Original version (german)**

Liebe Studienteilnehmer*in. Sie werden sich nun in einem Tiefgaragensetting wiederfinden (Bild). Hier wird ein Farbpunkt erscheinen (Bild). Achten Sie auf die Farbpunkte. Die unterschiedlichen Farbpunkte zu Beginn jedes Durchgangs geben an, wie nah die Bedrohung ist. Diese leuchten/ linken unterschiedlich schnell (blau = unpredictable, schwarz = predictable, je schneller desto näher, gelb = sicher/ keine Gefahr (Bild). Anschließend können Ihnen diese beiden Angreifer in der Tiefgarage begegnen (Bild). Der Gegenüber trägt entweder eine Waffe oder ein Mobiltelefon. Waffe: Bedrohung, Telefon: keine Bedrohung (Bild). Treffen Sie den Gegenüber im karierten Shirt [Shirt Farbe Counterbalanced zwischen Studienteilnehmern] an und werden a) erschossen oder b) erschießen ihn fälschlicherweise (Mobiltelefon), werden Sie NICHT elektrotaktil stimuliert (Bild). Treffen Sie den gegenüber im schwarz-weißen Shirt an und werden a) erschossen oder b) erschießen ihn fälschlicherweise (Mobiltelefon), werden Sie eletrotaktil stimuliert (Bild). Ihre Aufgabe: Sie können sich vor dem Angreifer schützen, indem Sie möglichst schnell mit einem Tastendruck reagieren und einen Schuss abwehren. Sollten Sie zu langsam sein, werden Sie erschossen und es folgt ein elektrischer Reiz an Ihrer Hand. Hinweis: Der telefonierende Mann ist KEINE Bedrohung, daher müssen Sie sich NICHT schützen. Sollten Sie fälschlicherweise schießen (Tastendruck), erhalten Sie einen elektrischen Reiz. Während des Experiments werden Töne abgespielt, die Sie durch die Kopfhörer wahrnehmen werden. Bitte wenden Sie Sich nun an Ihre*n Experimentalleiter*in und geben die Aufgabe in eigenen Worten wieder. Sie können nun gerne Fragen stellen. […] Bereit? Als erstes folgen Sicherheitsfragen. Anschließend beginnen Sie mit einem Übungsblock (8 Durchgänge) und können nochmal Fragen stellen. Gefolgt von dem Experimentalblock. Bleiben Sie während des gesamten Experimentalablaufs ruhig auf der Stabilometrischen Kräfteplattform stehen. Versuchen Sie Ihre linke Hand so ruhig wie möglich zu halten. […] Vielen Dank für Ihre Teilnahme am Experiment. Nun folgen Abschlussfragen.

## Pre‑Experimental Rating Questions

In the following, you will be asked questions about the perceived threat probability for each opponent. (Im Folgenden werden Ihnen Fragen zu den Bedrohungswahrscheinlichkeiten des jeweiligen Gegenübers gestellt.) How high (in %) do you estimate the probability of receiving an electrotactile stimulation from this opponent? (Wie hoch ist die Wahrscheinlichkeit (in %) einer elektrotaktilen Stimulation bei diesem Gegenüber?) How close do you perceive the threat for this colour cue? (Wie nah schätzen Sie die Bedrohung bei dieser Farbe ein?)

## Post‑Experimental Rating Questions

In the following, you will be asked questions regarding your impressions during the experiment. Please give a number between 0 and 100. (Im Folgenden werden Ihnen Fragen bezüglich Ihren Eindrücken während des Experiments gestellt. Bitte geben Sie eine Zahl zwischen 0 und 100 an.) How stressful did you perceive this dot to be? (Wie stressig haben Sie diesen Punkt wahrgenommen?) Do you have experience with computer games (e.g., first‑person shooters such as Call of Duty, Battlefield)? (Haben Sie Erfahrung mit PC‑Spielen (z. B. Egoshooter wie Call of Duty, Battlefield)?) I felt emotionally involved in the experiment. (Ich hatte eine emotionale Bindung zum Experiment.) How aversive did you find the electrotactile stimulation? (Wie aversiv haben Sie die elektrotaktile Stimulation wahrgenommen?) It was important to me to avoid the electrical stimulus. (Es war mir wichtig, den elektrischen Reiz zu vermeiden.) How aversive did you find the blood scene? (Wie aversiv haben Sie die Blutszene empfunden?)

## Calibration Instructions

**Step 1** Perception Threshold A stimulus will now be delivered to your right hand (Im Folgenden wird ein Reiz an Ihre rechte Hand gesendet). Please indicate whether you perceived this stimulus (Bitte geben Sie an, ob Sie diesen wahrgenommen haben). Note: It may take several trials until you notice a stimulus (Hinweis: Es kann mehrere Durchgänge dauern, bis Sie etwas wahrnehmen). Participants were verbally asked: “Did you perceive a stimulus on your right hand?” („Haben Sie einen Reiz an Ihrer rechten Hand wahrgenommen?“), with the response options “Yes” / “No” (ja / nein). They were then instructed: “For the next stimulus, please click the button ‘STIMULUS’.” („Für den nächsten Reiz bitte auf den Button ‘REIZ’ klicken.“) Intensity started at 0.10 mA and was increased in 0.10 mA steps until a “Yes” response was given.

**Step 2** Unpleasantness Threshold We will now determine a stimulus intensity that is very uncomfortable but tolerable (Nun wird ein Reizwert ermittelt, welcher sehr unangenehm aber aushaltbar sein soll). Please rate each stimulus on a scale from 0 (harmless) to 10 (unbearable) (Bewerten Sie bitte jeden Reiz zwischen 0 (harmlos) bis 10 (nicht aushaltbar)). After each stimulus, they were asked: “How unpleasant was this stimulus?” („Wie unangenehm war dieser Reiz?“) and reminded: “For the next stimulus, please click the button ‘STIMULUS’.” („Für den nächsten Reiz bitte auf den Button ‘REIZ’ berühren.“) The intensity began at 1.0 mA and was increased in 0.5 mA steps until the participant gave a rating of “7” (“very uncomfortable but tolerable”).

## Supplementary Figure 1. Individualized Approach for Electrotactile Stimulation

We implemented a systematic approach tailored to identify each participant’s aversive shock threshold. Initially, participants rated the perception of tactile stimulation (mean = 0.50 ± 0.25 ampere, Supplementary Figure 1A). Subsequently, shock calibration was executed to assess the efficacy of threat induction through electrotactile stimulation. Participants were tasked with providing threat ratings on a 0 to 10 scale, where 0 denoted non-aversive and 10 signified extremely aversive experiences (mean = 3.78 ± 2.37 ampere, Supplementary Figure 1B). Notably, within the defined range of 0-10, electrotactile stimulation targeted a level of 7, emphasizing its intentional placement as a moderately aversive stimulus. The perception threshold displays reduced individual variation, whereas a greater variation is observed in the experimental shock value (Supplementary Figure 1C).


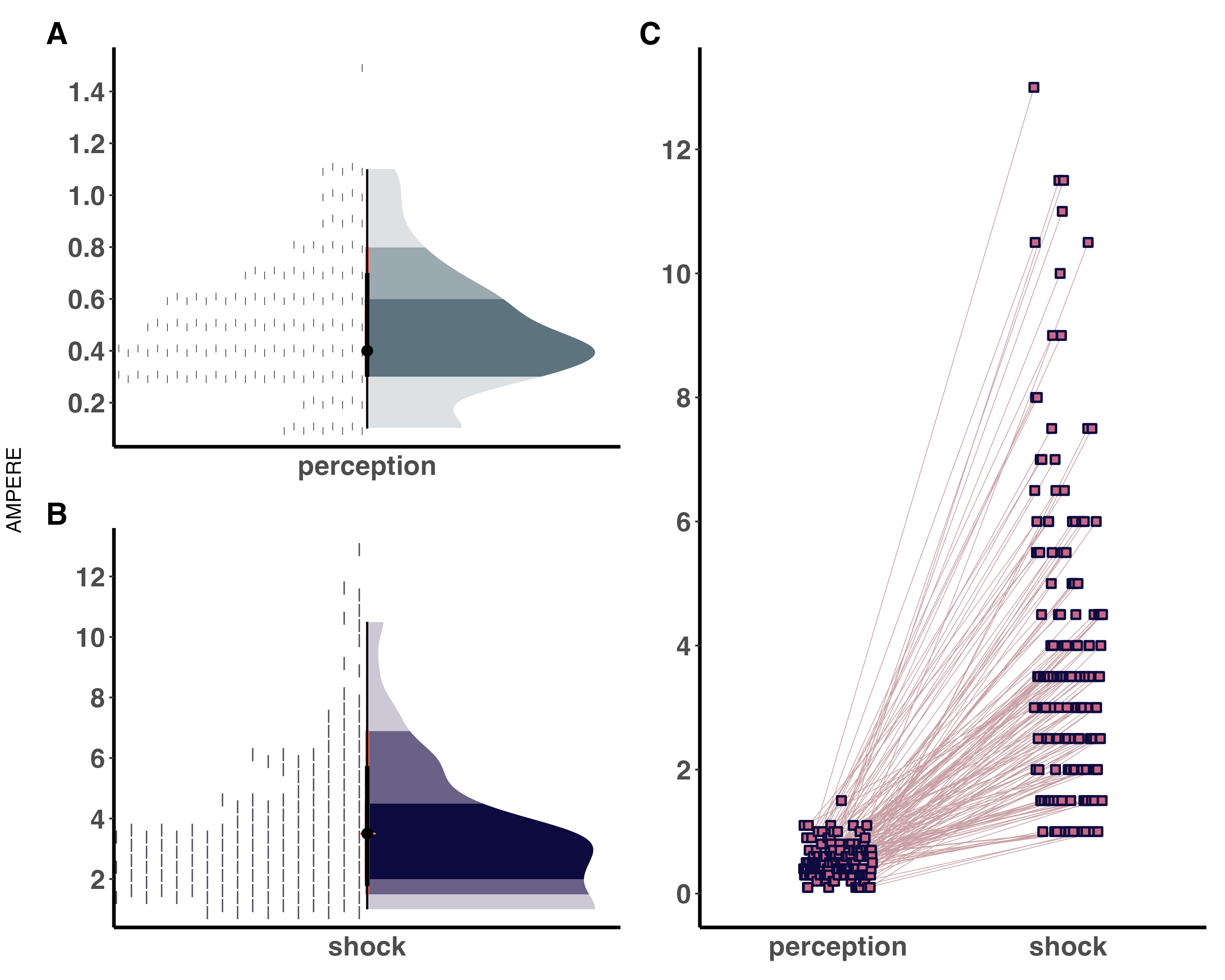


*Supplementary Figure* 1. The figure depicts individual perception thresholds (A) and experimental amplitudes of aversive electrotactile stimulation (B) with a line representing each individual and half-violin plots illustrating the group median and density. Furthermore, the relationship between individual perception values and experimental “shock” amplitudes is illustrated in C.

## Supplementary Table 1. Pairwise Comparisons for Threat-modulated Behavioral Performance


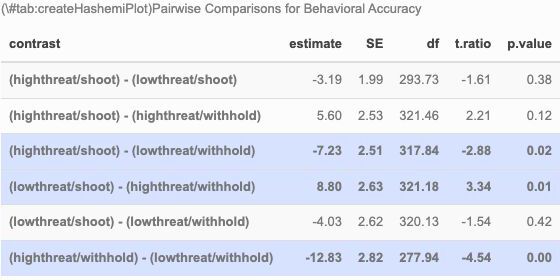


Supplementary Table 1. Pairwise Comparisons for Behavioral Accuracy.

## Supplementary Figure 2. Main Effect of Task in Postural Sway, Skin Conductance and Cardiac Responding


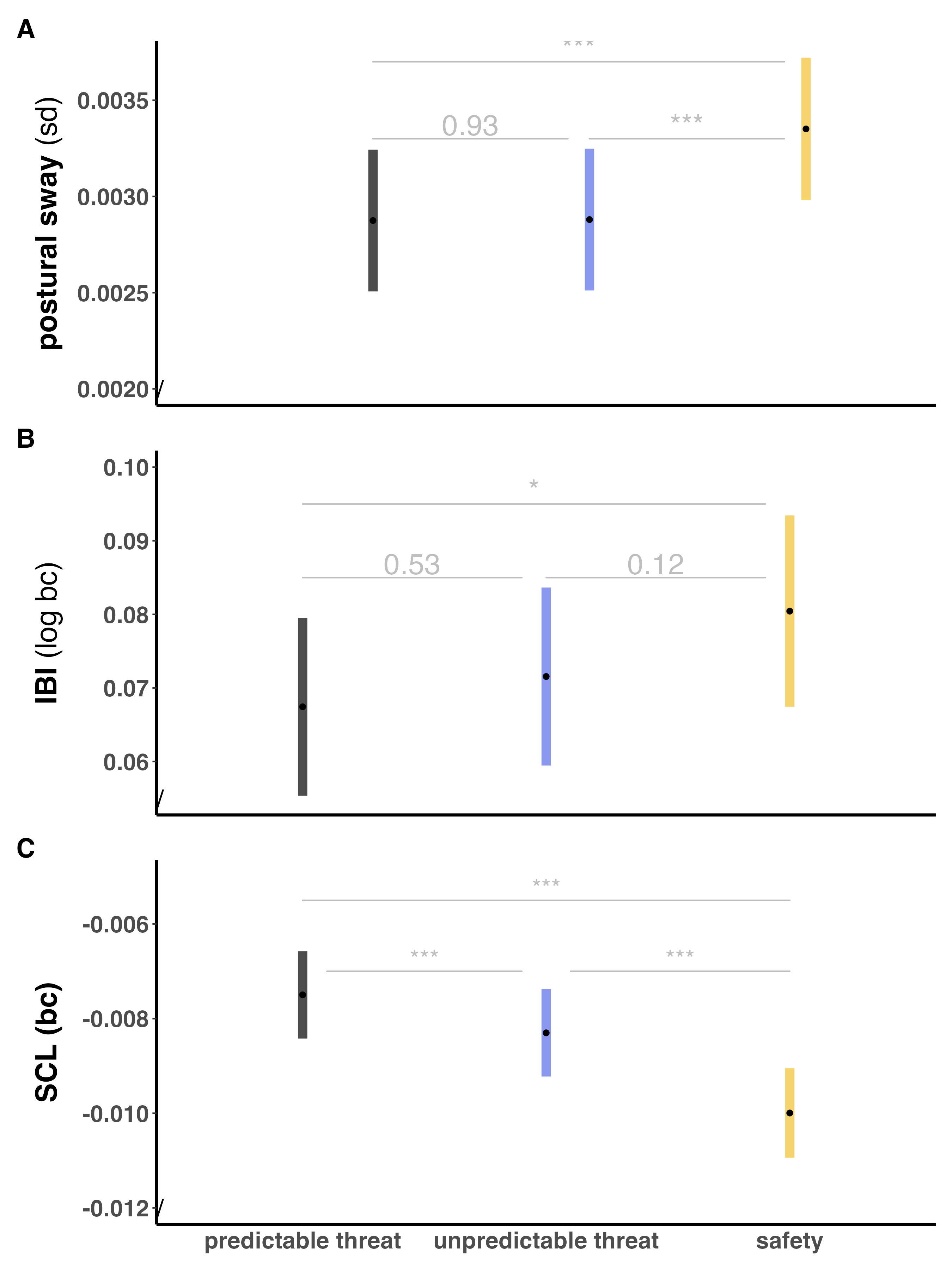


*Supplementary Figure* 2. Estimated marginal means with 95% confidence intervals illustrate postural sway (A), cardiac responding assessed by interbeat intervals (B), and tonic skin conductance levels during action preparation. The findings suggest a significant main effect of cue, with threat-induced postural freezing, cardiac freezing, and heightened skin conductance in both threat conditions as compared to the safety

## Supplementary Table 2. Pairwise Comparisons for the Interaction between Action Preparation and Time in Postural Sway


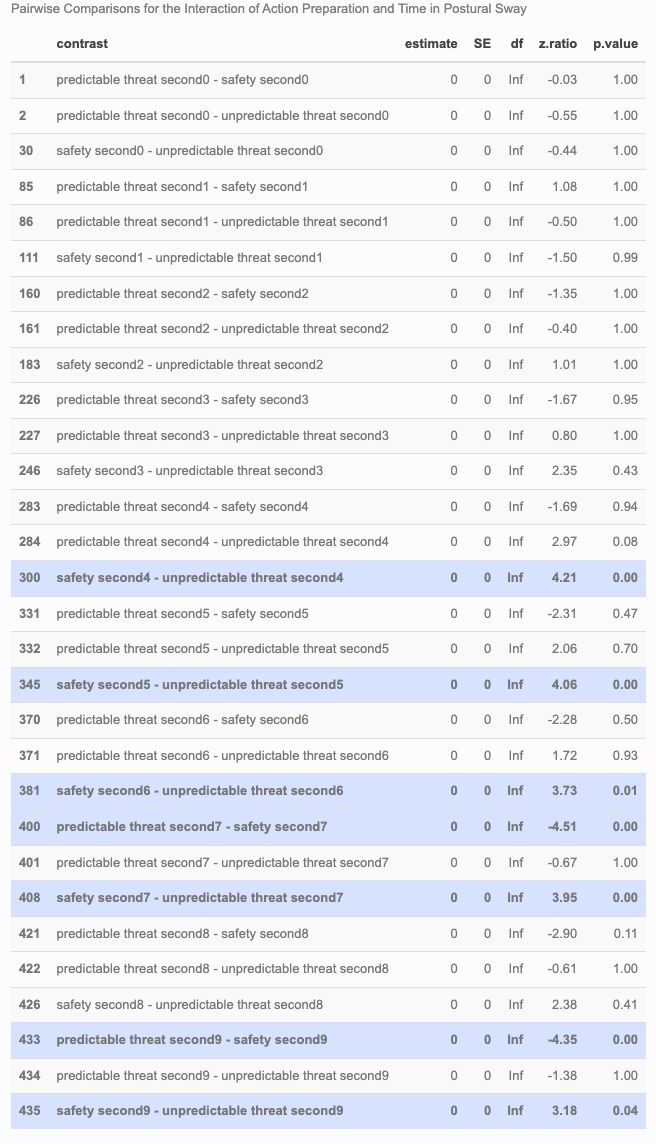


Supplementary Table 2. Multiple comparisons were Tukey-corrected.

## Supplementary Table 3. Pairwise Comparisons for the Interaction between Action Preparation and Time in Skin Conductance Levels


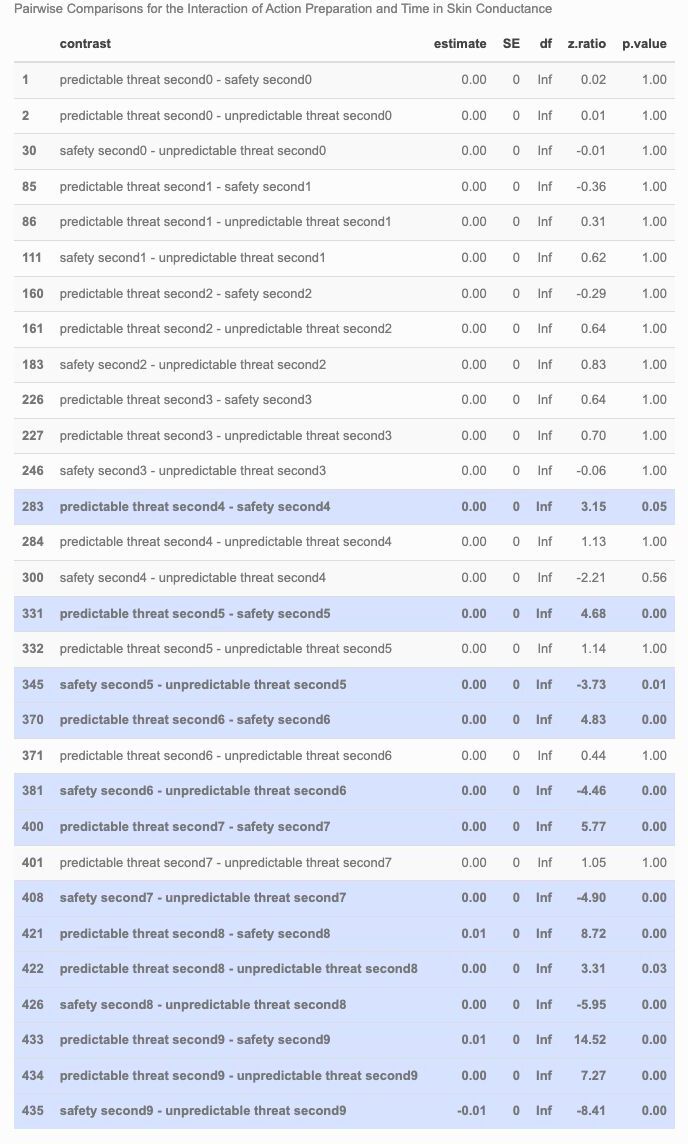


Supplementary Table 3. Multiple comparisons were Tukey-corrected.

## Supplementary Figure 3. Reaction time of ratings of anticipation stimuli (A) with visualization of individual differences (B) and reaction time of emotions (C)

Faster RT for providing the threat ratings were observed prior to the task as compared to after the task ([pre vs. post-task:] F(1, 1076) = 81.05, p < .001; i.e., main effect for time of assessment). Neither a significant main effect for cue (F(2, 965) = 1.18, p = 0.31), nor an interaction between cue and time of assessment was observed for RT (F(2, 965) = 1.3, p = 0.27, see Supplementary Figure 3A-C).


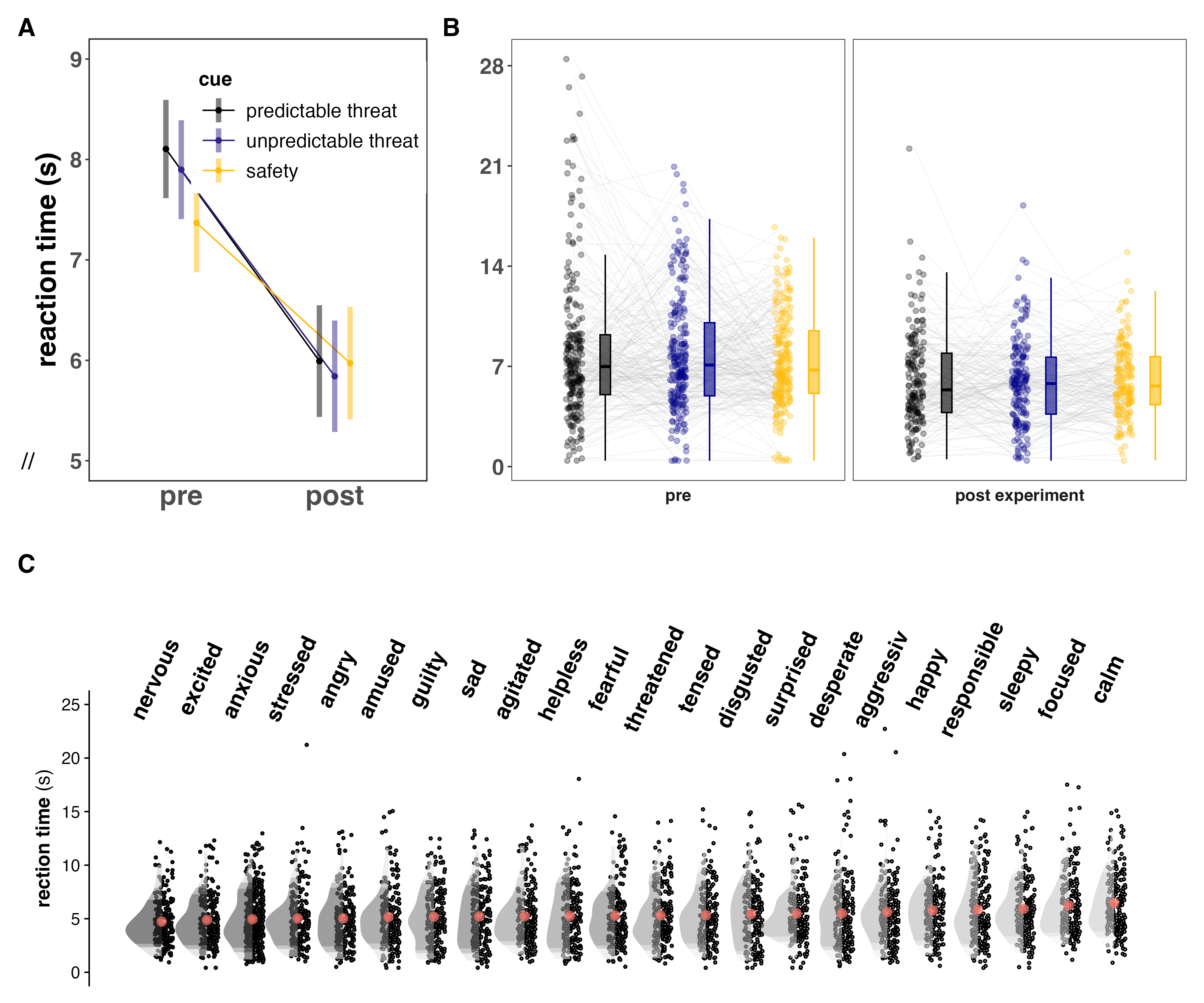


*Supplementary Figure* 3. Reaction times for threat ratings before and after the experiment, assessed on a visual analogue scale ranging from 0 (“not at all threatening”) to 100 (“extremely threatening”) for each condition, are presented. The figures include estimated marginal means with 95% confidence intervals (A) and boxplots with lines connecting dots to illustrate individual differences. Post-experimental reaction times for rating the subjective experience during the task are depicted using colored density plots for emotions, accompanied by dots representing each individual. Emotions are arranged in ascending order based on the sample mean of reaction time of experienced threat (red dots, C).

## Supplementary Figure 4. Rating of Avatars specific for each counterbalance condition


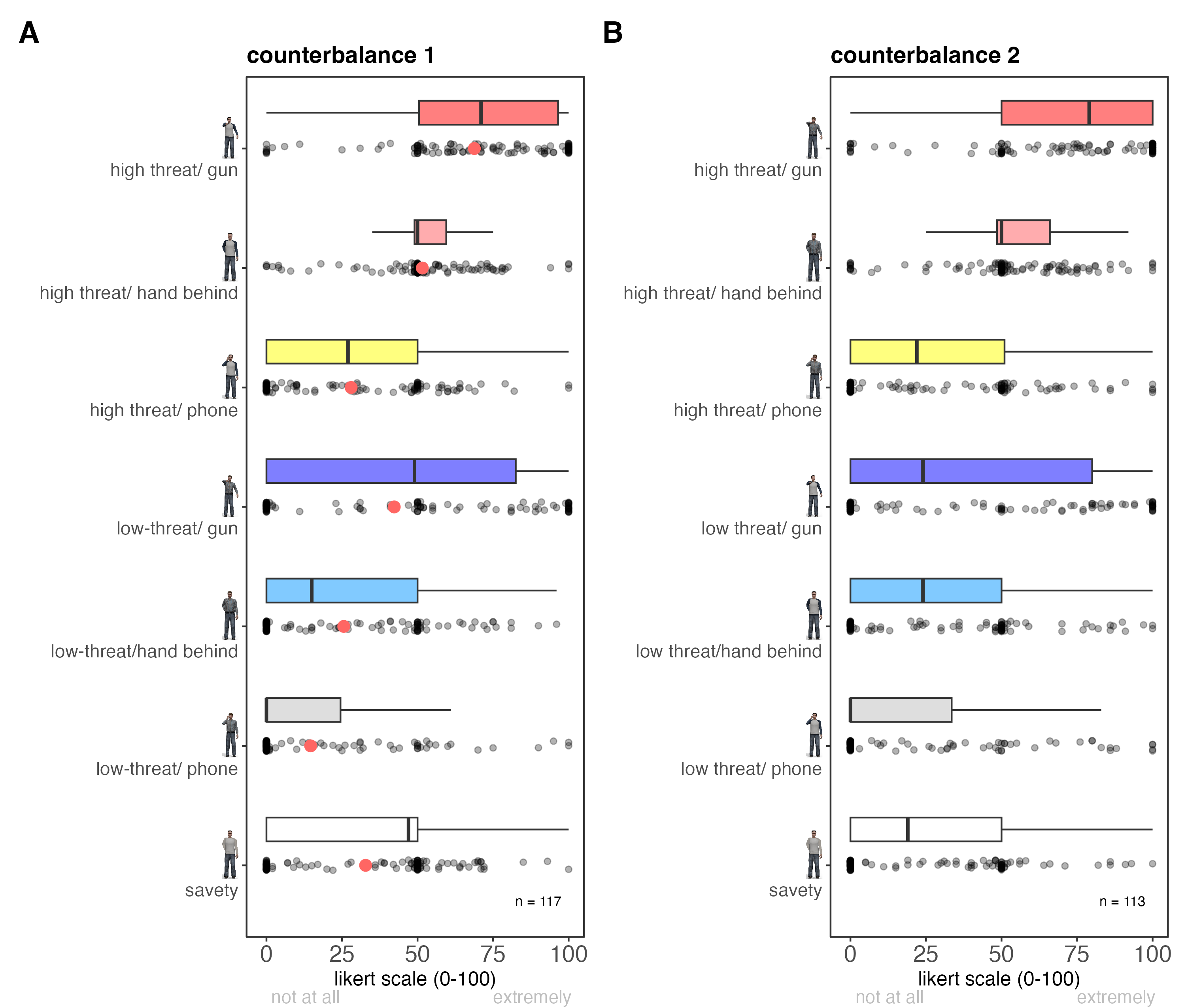


*Supplementary Figure* 4. Boxplots illustrate post-experimental ratings of avatars holding nothing, a phone, and a gun for counterbalance 1 (threat of shock associated with a black-white t-shirt avatar) and counterbalance 2 (threat of shock associated with a checkered t-shirt avatar). Linear mixed-effects models reveal a significant main effect for avatar (F(6,1407) = 45.68, p < .001), no significant main effect for counterbalance (F(1, 239) = 0.03, p = .86), and a significant interaction between avatar and counterbalance (F(6,1407) = 43.72, p < .001). This significant interaction of avatar and counterbalance was observed, indicating our intended threat manipulation - in both counterbalance conditions, the avatar associated with threat of shock was rated as the most adverse respectively, whereas avatars not associated with threat of shock were not. Reaction times for ratings did not demonstrate any significant main effect or interaction (all p’s > .24).

## Supplementary Figure 5. Post experimental Rating of motivation, aversiveness of shock, gaming experience and emotional attachment to the task


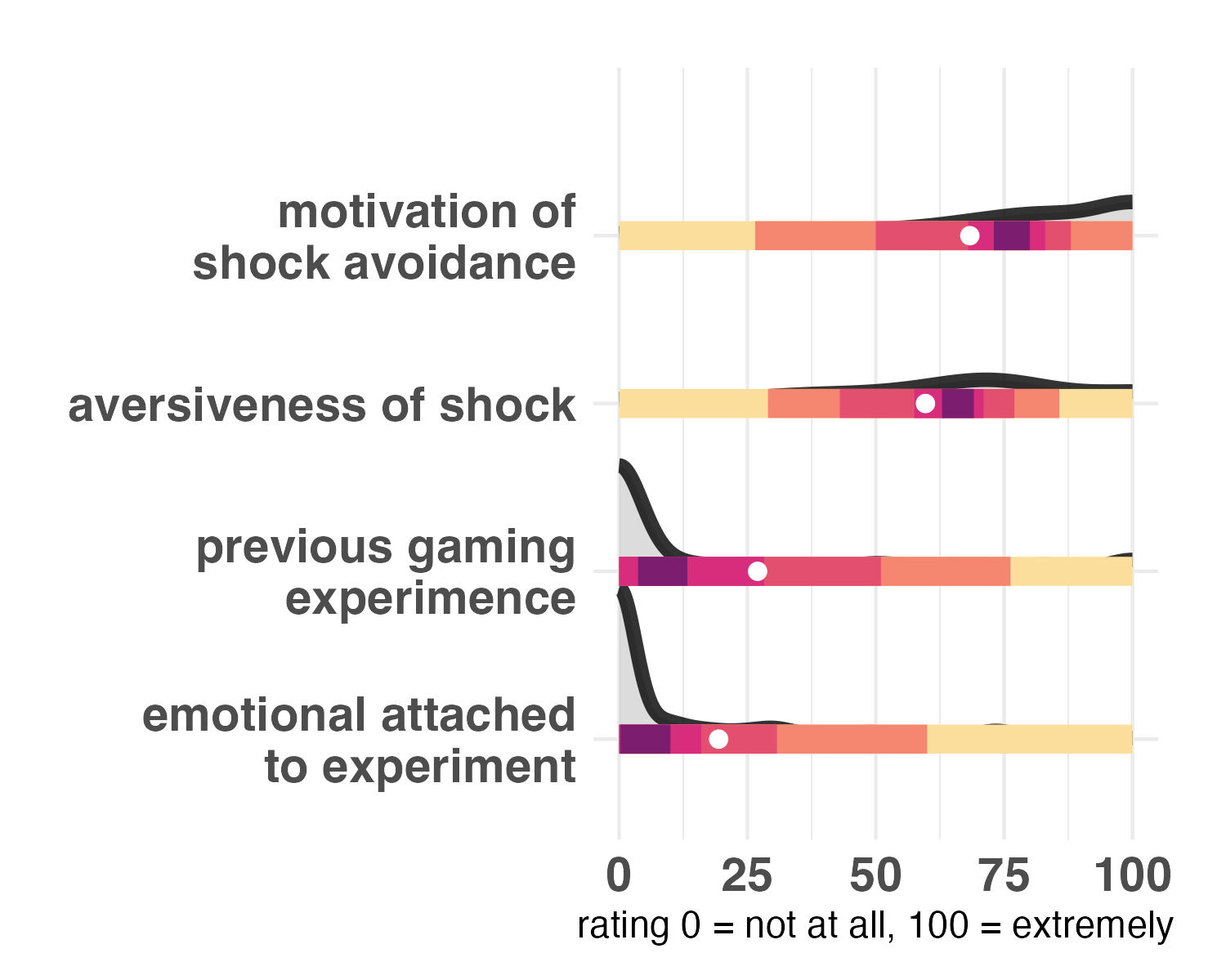


*Supplementary Figure* 5. Interval-Density Plots depict, on average, the high aversiveness of the electrotactile stimulation and the motivation to avoid it in the post-experimental ratings. Additionally, participants, on average, exhibited low gaming experience and emotional attachment to the experimental task.

## Data Preprocessing - Skin Conductance Responding

Skin conductance response data were down-sampled to a frequency of 10 Hz, and the responses were quantified offline while maintaining blindness to the type of stimulus using Brain Vision Analyzer, following established guidelines ([Boucsein, 2012](#ref-boucsein2012d)). Specifically, the trough was identified within an onset latency window ranging from 0.9 to 4 seconds after the onset of the stimulus (startle probe), while the peak was detected in a peak detection window (PDW) subsequent to response onset, lasting up to 5 seconds following the determined trough. For instances where multiple peaks were detected within the PDW, the first detected peak was selected. Each response identified by the algorithm underwent visual inspection and was manually adjusted when deemed necessary (for instance, in cases where the foot or trough was misclassified). Data points were range corrected based on the individuals maximum response and classified as missing when recording artifacts were present or when there was excessive baseline activity (i.e., greater than half of the response amplitudes) and were consequently excluded from subsequent analyses. Responses with an amplitude below a minimum threshold of 0.01 μS (for a justification see Lonsdorf et al., 2019) or where no response was observed (i.e., a flat line or habituation drift) within the specified time frame were categorized as non-responses (i.e., zero) and were included in the analyses.

## Supplementary Figure 6. No Startle-Modulation on Skin Conductance Responding

Additionally, we explored whether the phasic skin conductance response (SCR) was modulated by startle probes as reactions to the startle probe might interfere with the skin conductance level results presented above. No significant difference in SCRs elicited by startle probes administered during action preparation phase and the ITI (F(1,12695) = 1.6, p = .2, see Supplementary Figure 6) were observed and no significant differences between the cue conditions (predictable and unpredictable threat, safety) were observed (F (2,6152) = .3, p = .77, Supplementary Figure 6B).

*Supplementary Figure* 6. The estimated marginal means, along with 95% confidence intervals, depict phasic skin conductance responses (range corrected) following startle probes during action preparation as compared to the ITI (A). Additionally, Supplementary Figure 6B illustrates these responses for each cue condition. The results indicate that there is no significant effect of startle modulated skin conductance response between the threat anticipation conditions and/or the ITI.
